# Tachykinin-like NLP-58 signaling through TKR-1 modulates thermotaxis behavior in *Caenorhabditis elegans*

**DOI:** 10.1371/journal.pgen.1012346

**Published:** 2026-11-11

**Authors:** Moon Sun Jang, Hironori J. Matsuyama, Ryoga Suzuki, King Chuen Law, Yukimasa Shibata, Xiaolin Zhang, Koutarou D. Kimura, Yukihiko Kubota, Masahiro Ito, Kiyoji Nishiwaki, Ikue Mori

**Affiliations:** 1 Neuroscience Institute, Graduate School of Science, Nagoya University, Nagoya, Aichi, Japan; 2 Graduate School of Science, Nagoya City University, Nagoya, Aichi, Japan; 3 Department of Data Science, Faculty of Information Engineering, Toyama Prefectural University, Toyama, Japan; 4 Department of Bioinformatics, College of Life Sciences, Ritsumeikan University, Kusatsu, Shiga, Japan; 5 Department of Bioscience, Kwansei Gakuin University, Sanda, Japan; 6 Beijing Institute for Brain Research, Chinese Academy of Medical Sciences & Peking Union Medical College, Beijing, China; 7 Chinese Institute for Brain Research, Beijing, Beijing, China; University of Pennsylvania, UNITED STATES OF AMERICA

## Abstract

Neurokinin B (NKB) signaling plays a central role in mammalian thermoregulatory dysfunction associated with menopause, yet the underlying neural mechanisms remain incompletely understood. Here, we establish *Caenorhabditis elegans* thermotaxis as a genetically tractable system for investigating conserved tachykinin signaling. We demonstrate that overexpression of *nlp-58*, the *C. elegans* ortholog of mammalian NKB, induces abnormal thermotaxis behavior without impairing the temperature response of the thermosensory neurons. NLP-58 is expressed in multiple neurons, including the AIY interneurons, a central integrative node of the thermosensory circuit. AIY-specific overexpression of *nlp-58* is sufficient to reproduce the thermotaxis defect, identifying AIY as an important site of NLP-58-dependent behavioral modulation. Genetic analyses further demonstrated that the thermotaxis behavior is mediated primarily through the tachykinin receptor TKR-1, the homolog of mammalian tachykinin 3 receptor (NK3R), acting in ASG and/or ASJ sensory neurons. We also identified a genetic interaction between *dhs-4*, a lipid metabolic enzyme gene, and *nlp-58*, suggesting that lipid metabolic pathway modulate tachykinin-dependent thermotaxis behavior. Furthermore, endogenous NLP-58/TKR-1 signaling contributed to the age-dependent decline in thermotaxis behavior. Finally, treatment with fezolinetant, a selective NK3R antagonist used clinically for menopausal vasomotor symptoms, partially improved the thermotaxis defect caused by *nlp-58* overexpression. Together, our findings identify the NLP-58/TKR-1 signaling pathway as a modulatory component of thermotaxis behavior and provide a framework for investigating conserved principles of tachykinin-dependent circuit modulation.

## Author summary

Neurokinin B (NKB) signaling has emerged as a major regulator of menopausal vasomotor symptoms, but understanding how this neuropeptide modifies neural circuits remains challenging because of the complexity of mammalian nervous and endocrine systems. Here we used the simple nervous system of *Caenorhabditis elegans* to investigate conserved tachykinin signaling mechanisms. We found that overexpression of *nlp-58*, the worm ortholog of mammalian NKB, causes abnormal temperature preference behavior. Overexpression of *nlp-58* specifically in AIY interneurons was sufficient to reproduce this behavioral defect, and genetic analyses identified the tachykinin receptor TKR-1 as a major mediator of NLP-58 signaling. Expression of TKR-1 in either ASG and/or ASJ was sufficient to restore the behavioral effect of NLP-58 overexpression. We further show that endogenous NLP-58/TKR-1 signaling contributes to age-dependent decline in thermotaxis behavior. In addition, fezolinetant, an NK3R antagonist used clinically to treat menopausal vasomotor symptoms, partially suppressed the behavioral defect caused by *nlp-58* overexpression. Together, our findings establish *C. elegans* thermotaxis as a genetically and pharmacologically accessible system for investigating tachykinin-dependent modulation of temperature-related behavior and provide insights into conserved neuropeptide signaling mechanisms relevant to mammalian NKB signaling.

## Introduction

Menopause is a physiological transition characterized by declining ovarian function and reduced circulating estrogen levels. One of the most common and distressing symptoms during this period is hot flashes: sudden sensations of heat often accompanied by flushing and sweating. These vasomotor symptoms affect over 70% of menopausal women and significantly impact quality of life and daily functioning [1–5]. Although hormone replacement therapy remains the most effective treatment, concerns about its long-term safety, including increased risks of cardiovascular disease and breast cancer, have prompted the search for alternative therapeutic targets [6,7].

Accumulating evidence from human studies and rodent models has identified neurokinin B (NKB), a member of the tachykinin peptide family, as a key mediator of vasomotor symptoms. Following estrogen withdrawal, NKB expression is markedly upregulated in the hypothalamus, and exogenous NKB administration in premenopausal women elicits hot flash-like symptoms [8–14]. These findings indicate that estrogen withdrawal is associated with enhanced NKB signaling, which in turn contributes to thermoregulatory dysfunction. Nevertheless, elucidating the molecular and neuronal mechanisms underlying this pathway in mammals remains challenging due to the complexity of hormonal feedback networks, limited experimental access to specific neural circuits, and difficulties in quantitatively assessing thermoregulatory response.

A genetically tractable model system for dissecting conserved tachykinin signaling mechanisms would facilitate mechanistic understanding [15–17]. *Caenorhabditis elegans* offers several advantages for studying neuroendocrine regulation of behavior: its nervous system is fully mapped, genetically tractable, and amenable to single-cell level manipulation and analysis. Importantly, *C. elegans* exhibits robust thermosensory response, in which animals migrate along temperature gradients toward their prior experienced cultivation temperatures, providing a well-established and quantitative paradigm for investigating temperature-driven behavioral responses [18–21]. Previous works have identified core components of the thermosensory circuit, including the AFD thermosensory neurons and the AIY interneurons, which integrate thermal information and encode temperature preference [19,22,23]. Previous studies have suggested that steroid-related signaling pathways influence thermotaxis behavior in *C. elegans* [24].

In this study, we used *C. elegans* thermotaxis to investigate the conserved function of the tachykinin-like peptide NLP-58 and its receptor TKR-1. We demonstrate that overexpression of *nlp-58* induces abnormal, temperature-dependent thermotaxis behavior without disrupting primary thermosensory input, consistent with altered circuit modulation rather than sensory impairment. This phenotype is influenced by lipid metabolic pathways, revealing a functional link between neuropeptide signaling and lipid metabolism. Furthermore, through cell-specific overexpression and targeted rescue experiments, we identify the neurons that mediate NLP-58–dependent behavioral modulation and demonstrate that TKR-1 functions in specific sensory neurons that interface with the canonical thermosensory circuit.

Together, our findings uncover a conserved neuroendocrine mechanism linking lipid metabolism and temperature preference and establish *C. elegans* as a genetically tractable model for dissecting conserved neuropeptide signaling pathways relevant to mammalian neurokinin biology.

## Results

### Overexpression of NLP-58 results in thermotaxis behavioral defect

*C. elegans* exhibits typical thermotaxis behavioral pattern in consequence with its previous well-fed cultivation temperature. When animals are cultivated at well-fed conditions, they migrate toward the temperature at which they were previously cultivated, whereas starved animals lose this preference [18,19]. This experience-dependent thermotaxis allows the worms to stay near their previous cultivation temperature, representing an adaptive behavioral strategy that promotes survival under fluctuating environmental conditions (Fig 1A).

**Fig 1:**
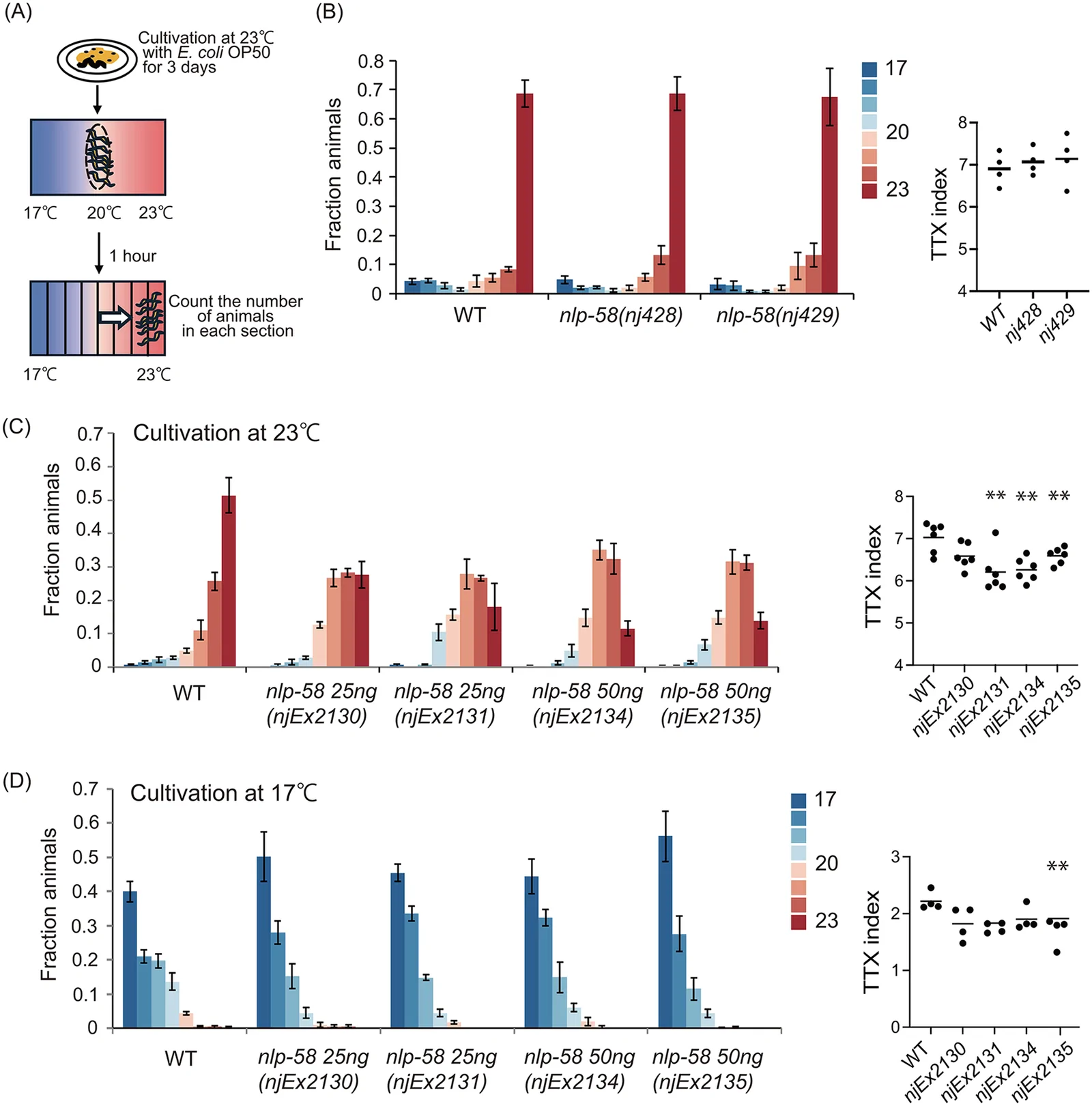
Overexpression of *nlp-58* induces abnormal thermotaxis behavior. (A) Schematic of the thermotaxis behavioral assay. Animals were cultivated at 23℃ for 3 days, transferred to a temperature gradient plate (17–23℃), and the number of animals in each section was counted after 1h. (B) Thermotaxis behavior of wild-type (WT) and *nlp-58* deleted mutants. Distributions of animals in each section of the plates are shown as mean ± SEM (standard error of the mean). TTX indices are presented as individual data points, with lines indicating the mean across independent experiments (n = 4). (C-D) Thermotaxis behavior of wild-type (WT), *nlp-58* overexpressing (25ng or 50ng genomic *nlp-58* expression) animals. Distributions of animals in each section of the plates are shown as mean ± sem. TTX indices are presented as individual data points, with lines indicating the mean across independent experiments. Animals cultivated at 23℃ were tested in independent assays (n = 4) (C) while those cultivated at 17℃ were tested in six independent assays (n = 6) (D). **p < 0 .01 one-way ANOVA with Dunnett’s multiple comparisons test with WT as the baseline.

To determine whether *nlp-58*, the *C. elegans* homolog of neurokinin B, is involved in thermotaxis behavior, we examined *nlp-58(nj428)* and *nlp-58(nj429)* mutants, in which exons of *nlp-58* were deleted, as well as *nlp-58* overexpression (OE) animals. Animals carrying a deletion of *nlp-58* exhibited normal thermotaxis, comparable to the wild type (Fig 1B). In contrast, *nlp-58*OE animals showed abnormal thermotaxis behavior when cultivated at 23 °C (Fig 1C). This behavioral defect was not observed when *nlp-58*OE animals were cultivated at 17 °C (Fig 1D). The absence of behavioral defects in *nlp-58* loss-of-function mutants, contrasted with the pronounced phenotype observed upon overexpression, indicates that NLP-58 is not an essential component of the thermosensory circuit but that proper regulation of its expression is critical for normal thermotaxis behavior.

In addition, *nlp-58*OE animals also exhibited several physiological abnormalities compared with the wild type. Adult day 1 *nlp-58*OE animals were significantly smaller than wild-type animals (S1A-S1B Fig). Furthermore, *nlp-58*OE animals laid significantly reduced number of eggs during a 20-hours period (S1C Fig). Regarding the defecation cycle, wild-type, *nlp-58*OE or deletion mutant animals all exhibited a gradual lengthening of the cycle with aging. In addition, the *nlp-58*OE animals tended to show a further prolongation of the defecation interval in aged animals, although this difference was not statistically significant (S1D Fig). In addition, *nlp-58*OE animals displayed a modest developmental delay, with a larger proportion of animals remaining at the L4 stage 52h after hatching compared with wild-type animals (S1E Fig). Importantly, despite these physiological differences, *nlp-58*OE animals exhibited normal locomotion speed (S1F Fig).

To determine whether *nlp-58* overexpression affects other sensory behaviors in addition to thermotaxis, we examined salt chemotaxis learning, another sensory-guided behavioral paradigm. *C. elegans* acquires a preference for the NaCl concentration experienced in the presence of food; for example, animals conditioned on plates containing 25 mM NaCl with food migrate toward this preferred salt concentration on a salt gradient assay plate, reflecting associative learning between food availability and salt concentration [25–27]. Like wild-type animals, *nlp-58*OE animals migrated toward the preferred salt concentration, and their chemotaxis index was not significantly different from that of wild-type animals. However, *nlp-58*OE animals showed greater inter-individual variability in their responses, with a tendency to move slower at the preferred salt concentration than wild-type animals (S1G Fig). These results suggest that *nlp-58* overexpression does not markedly impair salt-associated learning, although subtle effects on chemotactic performance cannot be excluded.

### Interaction between lipid metabolic enzyme DHS-4 and neuropeptide signaling in thermotaxis regulation

In mammals, NKB expression is suppressed by estrogen [28]. Our previous report demonstrated that estradiol modulates thermotaxis behavior in *C. elegans.* When estradiol was supplemented during cultivation, animals exhibited thermophilic behavior migrating toward temperatures higher than the cultivation temperature [24]. This effect is observed only in animals cultivated at 23 °C, but not at 17 °C. Under cultivation at 23 °C, expression of heat shock transcription factor HSF-1 is induced, leading to broad transcriptional changes associated with stress responses and metabolic adaptation. Among the HSF-1-regulated genes, we focused on *dhs-4*, which encodes a short-chain dehydrogenase/reductase involved in lipid metabolism, including estrogen biosynthesis and energy homeostasis [29]. To examine the role of *dhs-4* in thermotaxis regulation at 23°C, we analyzed *dhs-4* deletion mutants cultivated at 23 °C. These animals exhibited abnormal thermotaxis behavior: a substantial fraction of animals remained at lower temperatures (20.4 ~ 21.3 °C) instead of migrating toward their previous cultivation temperature, as observed in wild-type animals (Fig 2A). These findings suggest that, at 23 °C cultivation temperatures, HSF-1-dependent regulation of *dhs-4* expression level may contribute to estrogen-associated modulation of thermotaxis behavior, although the underlying molecular and neuronal mechanisms remain to be elucidated. The behavioral phenotype of *dhs-4* mutants closely resembled that of *nlp-58*OE animals. Because *dhs-4* participates in lipid metabolic processes, we hypothesized that *dhs-4*-dependent lipid intermediates may influence neuropeptide signaling pathways that regulate thermotaxis behavior. Given that estrogens are also lipid-derived metabolites synthesized from cholesterol, this relationship highlights a possible evolutionary connection between lipid metabolism and neuroendocrine mechanisms of thermoregulation.

**Fig 2:**
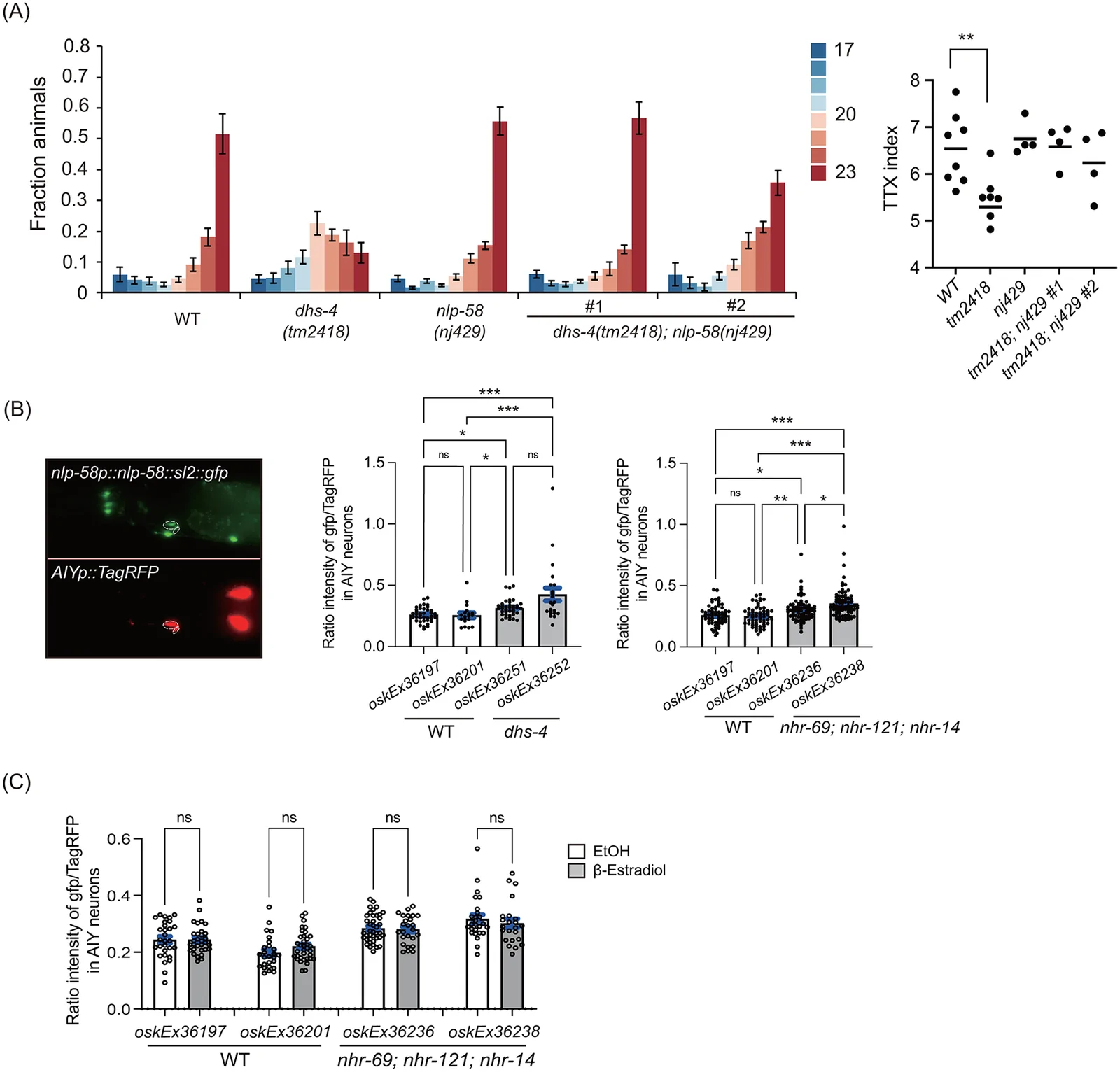
*nlp-58* genetically interacts with *dhs-4.* (A) Thermotaxis behavior of wild-type (WT), *dhs-4(tm2418)*, *nlp-58(nj429)*, and double mutant of *dhs-4* and *nlp-58(tm2418; nj429).* Animals were cultivated at 23℃ for 3 days. Distributions of animals in each section of the plates are shown as mean ± sem. TTX indices are presented as individual data points, with lines indicating the mean across independent experiments (n = 4 ~ 8). **p < 0.01 followed by one-way ANOVA with Tukey HSD multiple comparison test. (B) Comparison of *nlp-58* expression between WT and *dhs-4* or triple *nhr*-gene deleted background. Representative fluorescence images of animals expressing *nlp-58::GFP* driven by own promoter together with an AIY-specific TagRFP marker. The dotted line indicates the AIY interneurons (left). *nlp-58::GFP* fluorescence intensity was quantified and normalized to AIY-specific TagRFP. Box-and-whisker plots show the median (center line), interquartile range (box), and minimum to maximum values (whiskers). White bars represent wild-type animals, and gray bars represent three *nhr-*gene mutant animals. ns, not significant, *p < 0.05, **p < 0.01, ***p < 0.001 followed by one-way ANOVA with Tukey HSD multiple comparison test. (C) Quantification of *nlp-58::GFP* expression in AIY neurons following overnight treatment with 10 μM β-estradiol (gray bars) or vehicle control (EtOH) (white bars). Fluorescence intensity was quantified as the GFP/TagRFP ratio in AIY neurons. No significant differences were observed between β-estradiol- and vehicle-treated animals in either the wild-type or triple *nhr*-gene mutant background. Data are presented as mean ± SEM. Statistical significance was assessed using an unpaired two-tailed Student’s t-test. ns, not significant.

To test this possibility, we examined the genetic interaction between *dhs-4* and *nlp-58*. When cultivated at 23 °C, *dhs-4* deletion mutants displayed defective thermotaxis behavior, whereas deletion of *nlp-58* in the *dhs-4* deletion mutant background fully restored wild-type-like behavior (Fig 2A). These results indicate that *dhs-4* and *nlp-58* functionally interact in the regulation of thermotaxis behavior, suggesting that lipid metabolites produced by DHS-4 may modulate the neuropeptide-mediated thermosensory circuit. To further investigate the molecular mechanism regulating *nlp-58* expression, we examined candidate nuclear hormone receptor genes in *C. elegans*. Previous genetic studies implicated NHR-121, NHR-14, and NHR-69 in the regulation of thermotaxis behavior in *C. elegans* [24]. In addition, the estrogen-binding properties of these nuclear hormone receptors have been examined using a heterologous mammalian cell-based assay [30]. However, endogenous estrogen has not been established in *C. elegans*, and it remains unclear whether these receptors function as physiological estrogen receptors *in vivo*. We therefore analyzed *nlp-58* expression in *dhs-4* deletion mutants and in deletion mutants of *nhr-121*, *nhr-14*, and *nhr-69* nuclear hormone receptor genes. Notably, *nlp-58* expression was significantly elevated in *dhs-4* mutants and in *nhr-121*, *nhr-14*, and *nhr-69* mutants compared to wild-type animals (Fig 2B).

To determine whether exogenous estrogen directly regulates *nlp-58* expression, we treated animals with 10 μM β-estradiol following the protocol described previously [24]. β-estradiol treatment did not significantly change *nlp-58* expression under our experimental conditions (Fig 2C). These findings indicate that although *dhs-4* and the candidate nuclear hormone receptors genetically regulate *nlp-58* expression, our data do not support a simple model in which β-estradiol directly represses *nlp-58* transcription. Instead, these results suggest that *nlp-58* expression is regulated by steroid hormone-related signaling pathways or other lipid-derived metabolites whose identities remain to be determined.

### NLP-58/TKR-1 signaling contributes to age-dependent decline in thermotaxis behavior

Because *nlp-58*OE animals showed greater inter-individual variability in the age-related prolongation of the defecation cycle than wild-type animals (S1D Fig), we next asked whether endogenous NLP-58 signaling is also regulated during aging. To address this question, we examined age-dependent changes in the expression of components of the NLP-58 signaling pathway using RNA sequencing. Our analysis revealed that both *dhs-4* and *nlp-58* were expressed at relatively constant levels throughout aging. In contrast, among the three tachykinin receptor genes (*tkr-1, tkr-*2, and *tkr-3*), transcript levels of *tkr-1* and *tkr-2*, which are thought to encode known receptors for NLP-58, showed age-dependent increases in transcript abundance (Fig 3A). As a positive control, *cpi-1*, a gene previously reported to increase with aging, exhibited the expected age-dependent upregulation [31].

**Fig 3:**
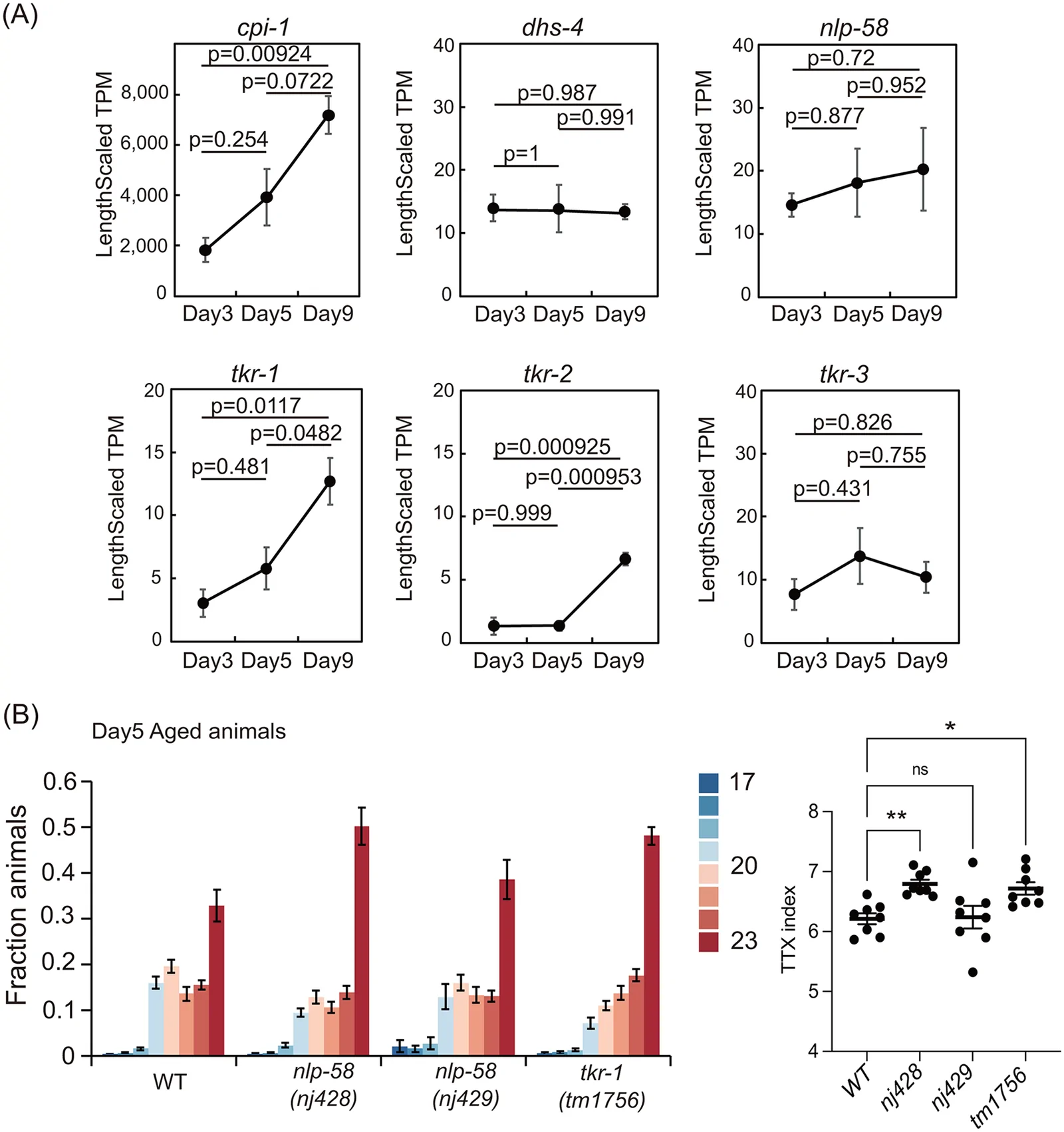
Age-dependent changes in NLP-58/TKR-1 signaling contribute to thermotaxis decline. (A) RNA-seq analysis showing age-dependent expression changes of *dhs-4*, *nlp-58*, *tkr-1*, *tkr-2*, and *tkr-3*. *cpi-1* served as an age-dependent positive control. P values were determined by one-way ANOVA with Tukey HSD multiple comparison test. (B) Thermotaxis behavior of Day 5 adult wild-type, *nlp-58(nj428)*, *nlp-58(nj429)*, and *tkr-1(tm1756)* animals. Left, distribution of animals across the thermal gradient. Right, thermotaxis index (TTX index). Data are presented as mean ± SEM. Statistical significance was determined using one-way ANOVA followed by Tukey’s multiple comparisons test. *P < 0.05, **P < 0.01; ns, not significant.

Previous studies have established that thermotaxis behavior declines with age in *C. elegans* [32–34]. Based on these findings, we asked whether NLP-58/TKR-1 signaling contributes to the impaired thermotaxis observed in aging animals. We therefore examined the thermotaxis behavior among aged wild-type, *nlp-58*, and *tkr-1* mutant animals. Compared with aged wild-type animals, aged *nlp-58 (nj428)* and *tkr-1(tm1756)* deletion mutants showed significantly improved thermotaxis indices, and while *nlp-58 (nj429)* mutants showed a similar trend, their thermotaxis index did not reach statistical significance (Fig 3B). These findings suggest that the NLP-58/TKR-1 signaling pathway contributes to impaired thermotaxis behavior in aged *C. elegans*.

### AIY-specific overexpression of *nlp-58* mimics thermotaxis abnormality of *nlp-58*OE animals

To understand where and how NLP-58 functions to regulate the thermal response, we examined the cellular expression pattern of *nlp-58* by expressing a genomic *nlp-58p::nlp-58::sl2::GFP::nlp-58 3’UTR* fusion in the wild-type background. Using the multi-colored NeuroPAL marker strain [35] and co-localization analysis with cell-specific fluorescent markers, we found that the *nlp-58::GFP* reporter is expressed in multiple neurons located in both the head and tail regions, as well as in several non-neuronal cells. In the head and tail regions, *nlp-58* expression was consistently observed in the four polymodal OLQ sensory neurons, as well as in PHC, FLP, ASG, AWB, and AVE neurons, with OLQ showing particularly strong expression. In contrast, weaker and more variable expression was detected across individuals in ASE, AVG, RIM, IL2, AVD, AVA, URX, AIN, AUA, ROB, AMso, AQR and AIY (Fig 4A). In the tail, *nlp-58* was robustly and reliably expressed in a single pair of PHC sensory neurons (Fig 4B). NLP-58 expression was also detected in neurons with long axonal projections spanning from the head to the tail. These neurons were not identified with confidence, however, because our analysis focused primarily on cells located in the head and tail regions. Co-localization analysis further revealed *nlp-58* expression in the AIY interneurons, which are key components of the thermosensory circuit (Fig 4C). However, NLP-58 expression in AIY neurons could not be reliably identified using the NeuroPAL strain under our standard cultivation condition of 23 °C likely due to the extremely low expression level at this temperature. When animals were cultivated at 15 °C, NLP-58 expression in AIY became more apparent and was markedly increased compared with 23 °C (S2 Fig and S1 Table). These results suggest that *nlp-58* expression in AIY is weak and highly sensitive to temperature.

**Fig 4:**
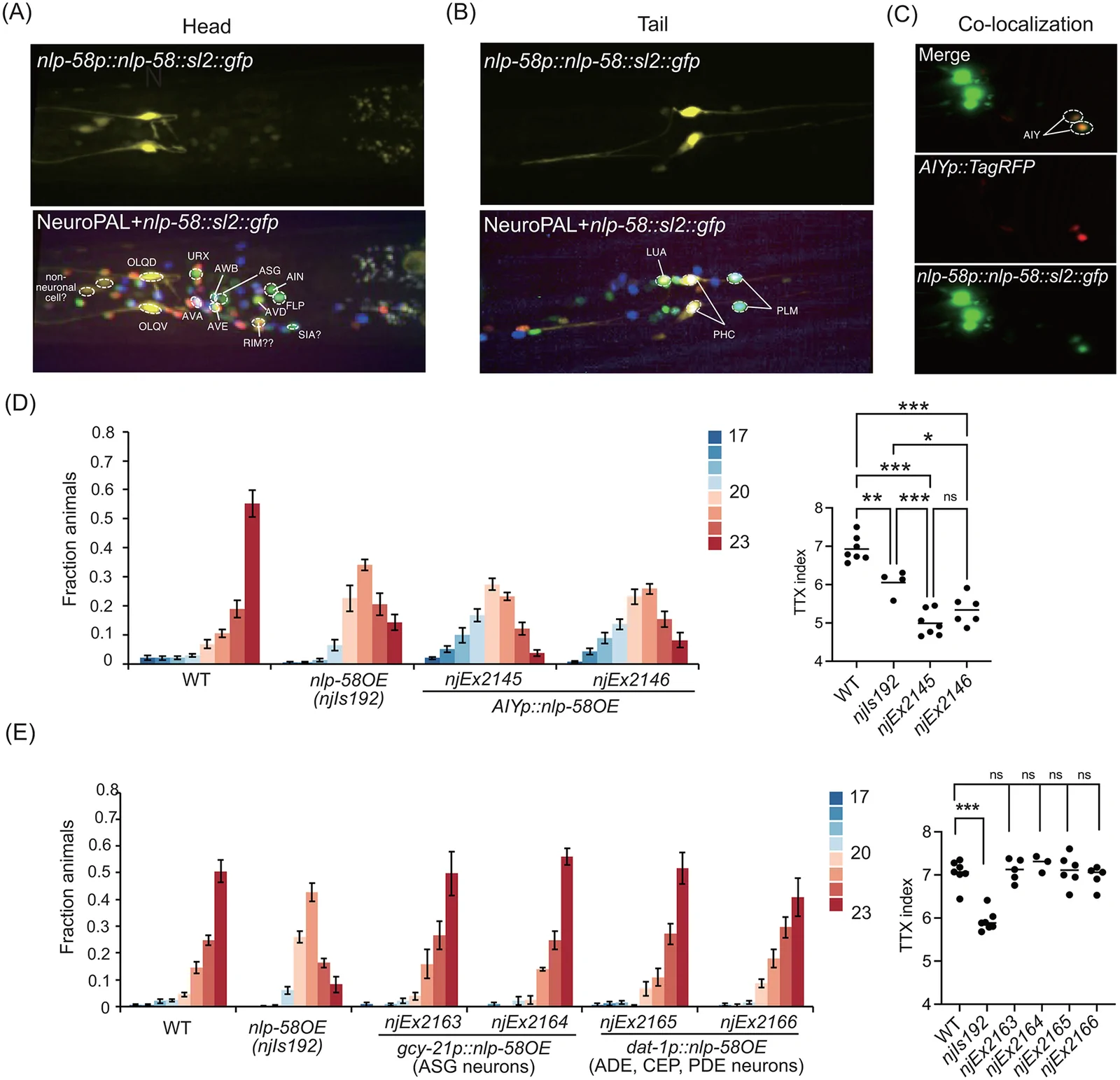
NLP-58 functions in AIY interneurons to regulate thermotaxis behavior. (A and B) Representative confocal micrographs showing the reporter (top) merged with multi-colored nuclei in the NeuroPAL strain (bottom) in head (A) and tail (B). (C) Co-localization analysis between the *nlp-58p::nlp-58::sl2::GFP* reporter and AIY-specific TagRFP reporter. (D) Thermotaxis behavior of wild-type (WT), *nlp-58*OE animals with the endogenous *nlp-58* promoter (*njIs192*), and with AIY-specific promoter (*njEx2145* and *njEx2146*). (E) Thermotaxis behavior of wild-type (WT), *nlp-58*OE animals with expression driven by the endogenous *nlp-58* promoter, with ASG- or dopaminergic neurons-specific promoters. Distributions of animals in each section of the plates are shown as mean ± SEM. TTX indices are presented as individual data points, with lines indicating the mean across independent experiments. *p < 0.05, **p < 0.01, ***p < 0.001, ns, not significant followed by one-way ANOVA with Tukey HSD multiple comparison test (C and D).

Based on the neuronal expression patterns of *nlp-58*, we next examined which cells are responsible for the abnormal thermotaxis behavior observed in the *nlp-58*OE animals. To this end, we overexpressed *nlp-58* in specific neurons where its endogenous expression was detected (ASG and AIY) or not detected (CEP and ADE) and tested thermotaxis behavior of the overexpression strains. We selected these neurons based on the expression pattern observed in our *nlp-58* reporter together with the availability of established cell-specific promoters. Interestingly, overexpression of *nlp-58* specifically in AIY interneurons was sufficient to induce the thermotaxis defect observed in the *nlp-58*OE animals, while animals overexpressing *nlp-58* in ASG, CEP, or ADE neurons exhibited normal thermotaxis behavior (Fig 4D and 4E).

AIY interneurons integrate inputs from AFD thermosensory neurons and transmit the processed signals to downstream motor circuits [19,36]. The finding that *nlp-58* overexpression in AIY recapitulates the phenotype of *nlp-58*OE animals implicates that NLP-58 secretion from AIY interneurons plays a critical role in regulating thermotaxis behavior.

### NLP-58 overexpression does not impair AFD thermosensory activity

AFD sensory neurons are the primary thermosensory neurons in *C. elegans* and are known to sense temperature changes during thermotaxis behavior [20,37]. AFD neurons are activated upon warming and inactivated upon cooling, and the onset temperature of their activity depends on the cultivation temperature [20,23]. To examine whether the abnormal thermotaxis phenotype caused by *nlp-58* overexpression results from impaired thermosensory input, we monitored AFD neuronal activity by expressing the calcium indicator GCaMP3 [38] specifically in AFD neurons and applying a linear temperature ramp from 17 °C to 25 °C. The temperature-dependent responses of AFD neurons were indistinguishable between wild-type and *nlp-58*OE animals (Fig 5A-5D). These results indicate that excessive *nlp-58* signaling does not affect AFD thermosensory function.

**Fig 5:**
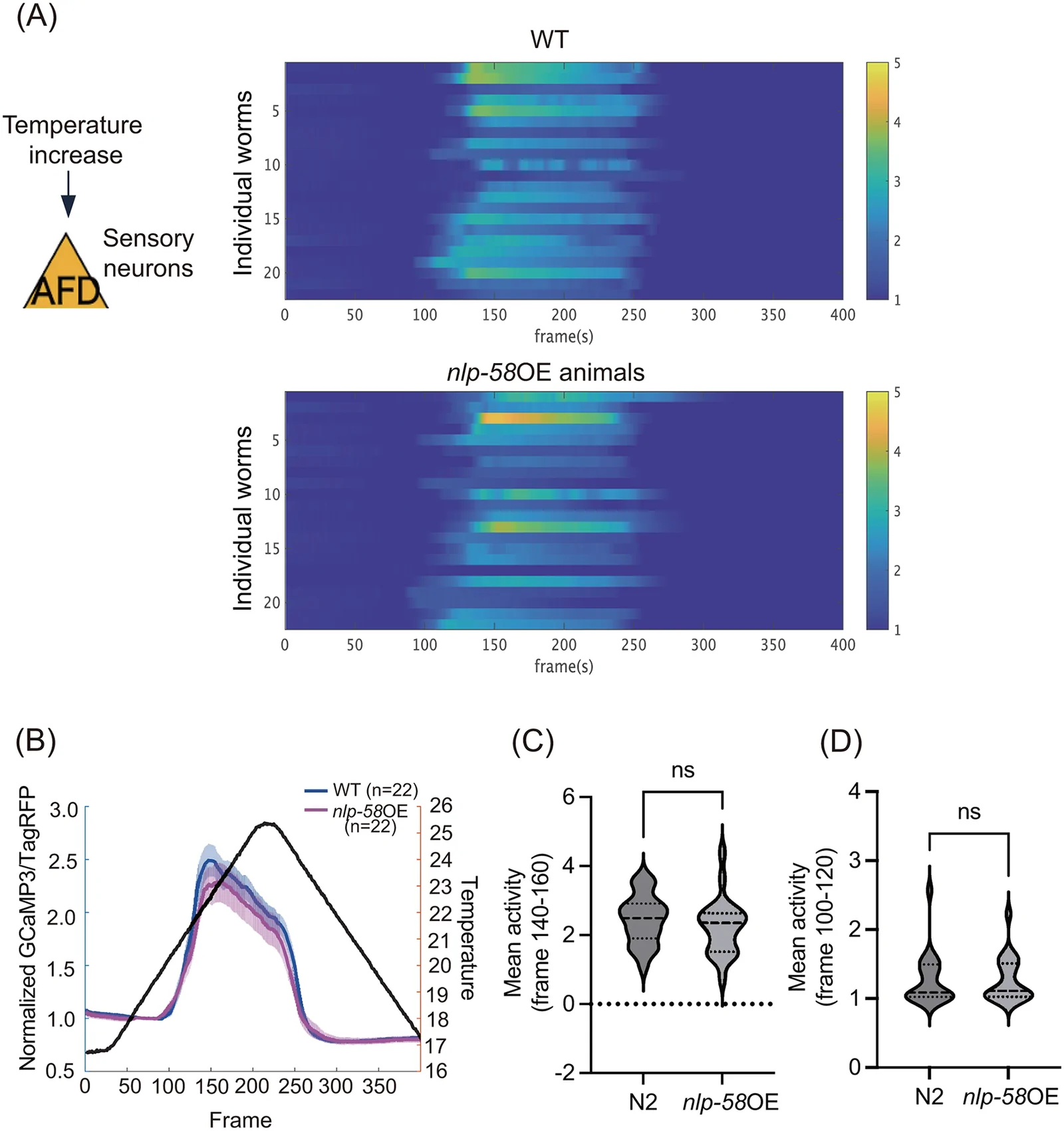
NLP-58 overexpression does not impair AFD thermosensory response. (A) Schematic of temperature stimulus and AFD neuron (left). Heat maps represent normalized ratio changes of AFD calcium dynamics using GCaMP3 in wild-type (WT) and *nlp-58*OE animals (*njIs192*) during a linear temperature ramp (17 °C to 25 °C). Each row represents the response of an individual animal. (B) Mean ± SEM of AFD responses in wild-type N2 (blue line) and *nlp-58*OE (red line) from animals each, with temperature stimulus shown (black line). (C and D) Violin plot of AFD activities during 140–160 s (C) or 100–120 s (D) after the thermal stimulus was applied. ns, not significant followed by unpaired two-tailed Student’s t-tests.

### TKR-1 plays a predominant role in *nlp-58*-dependent thermotaxis behavior

Previous studies using ligand-binding assays in mammalian cells identified TKR-1 and TKR-2 as functional tachykinin receptors mediating *nlp-58* signaling in *C. elegans* [39,40]. To further investigate genetic interaction among tachykinin receptors in the regulation of thermotaxis behavior, we analyzed mutants of the three receptor genes (*tkr-1, tkr-2,* and *tkr-3*) encoded in the *C. elegans* genome. Single and triple receptor mutants all exhibited normal thermotaxis behavior, indistinguishable from that of wild-type animals (Fig 6A).

**Fig 6:**
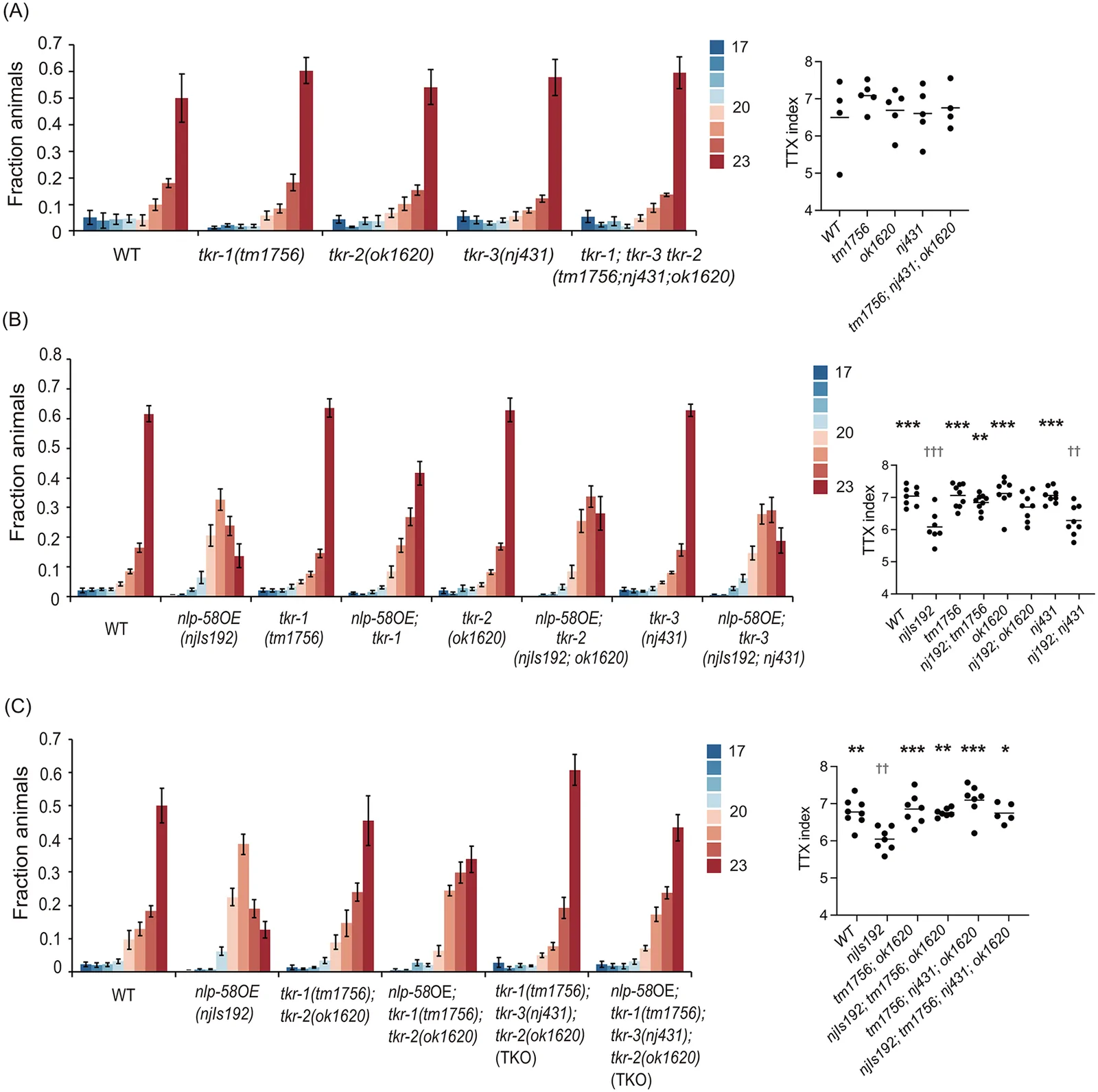
TKR-1 plays a predominant role in NLP-58-dependent thermotaxis behavior. (A) Thermotaxis behavior of wild-type (WT), *tkr-1 (tm1756), tkr-2 (ok1620), and tkr-3 (nj431)*, and triple mutants (TKO). ns, not significant followed by one-way ANOVA with Dunnett’s multiple comparisons test to WT. (B and C) Thermotaxis behavior of wild-type (WT), *nlp-58*OE animals, tachykinin receptor single mutant, and *nlp-58*OE animals carrying receptor deletions (B) and *nlp-58*OE animals combined with double or triple receptor deletions (C). ++p < 0.01, +++p < 0.001 from comparison with WT and *p < 0.05, **p < 0.01, ***p < 0.001 from comparison with *nlp-58*OE strain by one-way ANOVA with Tukey HSD multiple comparison test. Animals were cultivated at 23℃ for 3 days. Distributions of animals in each section of the plates are shown as mean ± SEM. TTX indices are presented as individual data points, with lines indicating the mean across independent experiments.

To determine whether these receptors mediate the abnormal behavior observed in *nlp-58*OE animals, we generated double mutants carrying *nlp-58*OE and mutations in tachykinin receptor genes. The thermotaxis defects of *nlp-58*OE animals were significantly suppressed by the *tkr-1* mutation, restoring thermotaxis behavior to a level comparable to wild-type animals. The *tkr-2* mutation produced only a modest suppression of the *nlp-58*OE phenotype, and *tkr-3* mutation did not show the suppression of the *nlp-58*OE phenotype (Fig 6B). Deletion of all three *tkr* genes significantly suppressed the *nlp-58*OE phenotype, resulting in a TTX index that was not significantly different from that of wild-type animals (Fig 6C). These findings indicate that tachykinin receptors contribute differentially to *nlp-58*-dependent thermotaxis behavior and that TKR-1 plays a predominant role. Consistent with this interpretation, CeNGEN single-cell RNA-seq datasets indicate that *tkr-1*, *tkr-2*, and *tkr-3* exhibit distinct cellular expression patterns (CeNGEN.org), supporting the possibility that these receptors function in different neuronal contexts. Together, our results indicate that multiple tachykinin receptors contribute to *nlp-58*-dependent thermotaxis behavior with TKR-1 making the largest contribution.

### TKR-1 in ASG or ASJ sensory neurons mediate *nlp-58* signaling in *nlp-58*OE animals

We next sought to identify the neuronal cell-type in which TKR-1 functions in the context of *nlp-58*–dependent signaling in thermotaxis behavior. Co-localization analysis revealed that *tkr-1* is expressed in ASG and ASJ sensory neurons, as shown by a *tkr-1* promoter–driven TagRFP reporter and dye-filling with DiO, which labels several sensory neurons including ASI, ADL, ASK, AWB, ASH, and ASJ (Fig 7A-7C).

**Fig 7:**
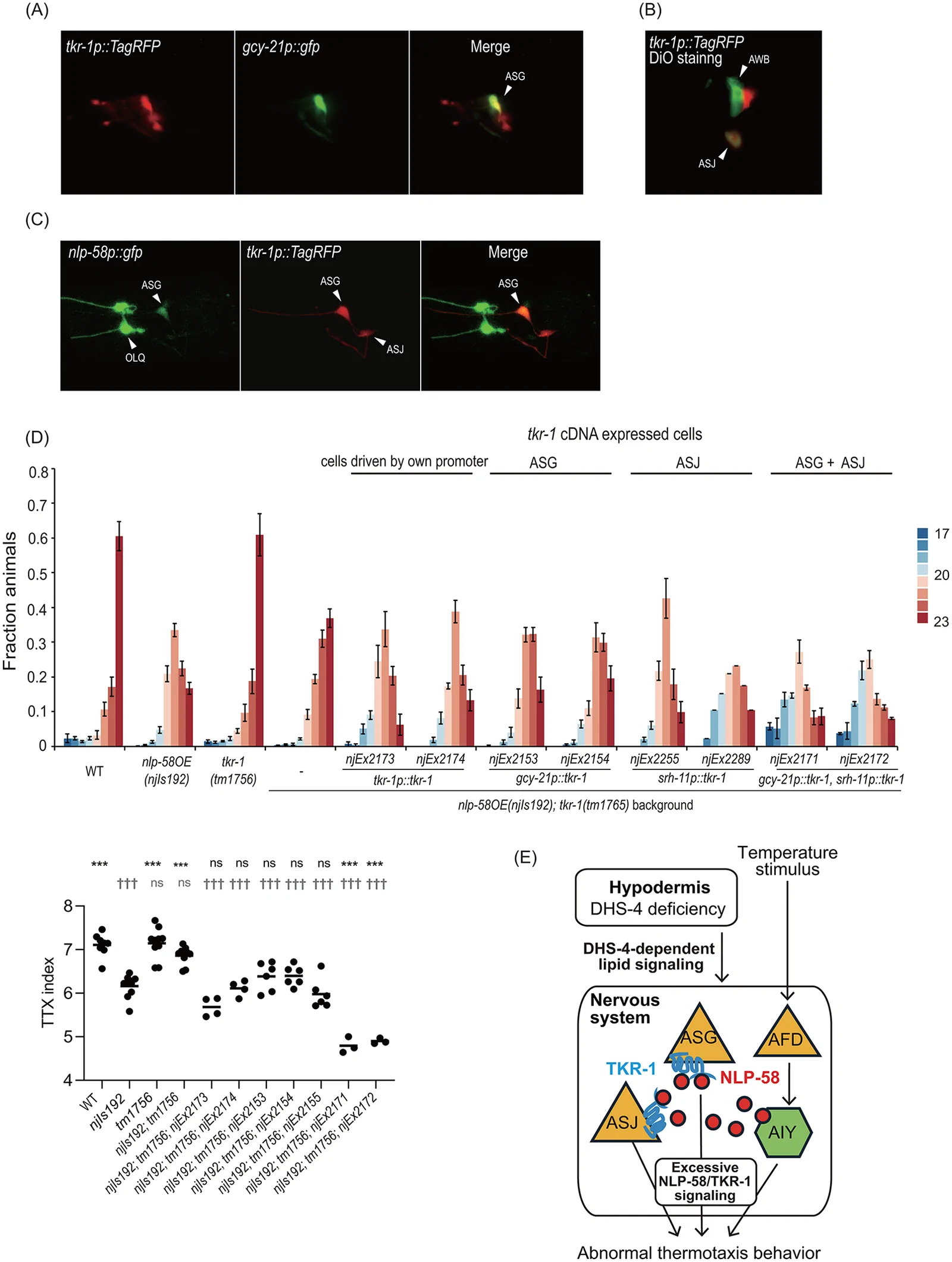
TKR-1 functions in ASG and ASJ neurons to mediate NLP-58-dependent thermotaxis regulation. (A) *tkr-1p::TagRFP* (left) was colocalized with ASG cell-specific GFP (right). (B) Cell identification of t*kr-1p::TagRFP* reporter in DiO dyed animals. The ASI, ADL, ASK, AWB, ASH, and ASJ amphid neurons in head are known to be dyed with DiO [62]. (C) Co-localization analysis between the *nlp-58p::GFP* and *tkr-1p::TagRFP*. (D) Thermotaxis behavior of wild-type (WT), *nlp-58*OE animals, *tkr-1(tm1756)* mutant, *nlp-58*OE animals with *tkr-1* gene deletion, and *tkr-1(tm1756)* mutant strains rescued by *tkr-*1 cDNA expression under its own promoter, in ASG neurons, in ASJ neurons, or in both ASG and ASJ neurons. ***p < 0.001 from comparison with *nlp-58*OE strain and +++p < 0.001 from comparison with WT by one-way ANOVA with Tukey HSD multiple comparison test. Distributions of animals in each section of the plates are shown as mean ± SEM. TTX indices are presented as individual data points, with lines indicating the mean across independent experiments. (E) Proposed model for thermosensory behavioral regulation by NLP-58/TKR-1 signaling.

Based on the expression pattern of *tkr-1*, we next performed cell-type-specific rescue experiments to identify the neurons in which TKR-1 acts to mediate *nlp-58* signaling. We showed that overexpression of *nlp-58* caused abnormal thermotaxis behavior, and that this behavioral defect was significantly suppressed by the *tkr-1* loss-of-function mutation (Fig 7D). To determine the neurons in which TKR-1 functions to mediate this effect, we expressed *tkr-1* specifically in ASG and/or ASJ neurons in *nlp-58*OE; *tkr-1* loss-of-function animals. Expression of *tkr-1* cDNA in either ASG or ASJ neurons was sufficient to restore the abnormal thermotaxis phenotype of *nlp-58*OE animals in the *tkr-1* mutant background. Simultaneous expression in both ASG and ASJ produced a stronger phenotype than expression in either neuron alone, suggesting that TKR-1 signaling in these neurons may have a combined effect on thermotaxis behavior. Together, these findings suggest that TKR-1 expression in either ASG or ASJ sensory neurons is sufficient to mediate the behavioral effects of NLP-58 overexpression.

Based on these findings, we propose the following model. In *nlp-58* overexpression animals, temperature stimuli are initially detected by the primary thermosensory neuron AFD and transmitted to the downstream AIY interneurons, as in the canonical thermosensory circuit. However, when NLP-58 is overexpressed and excessively secreted from AIY interneurons, this neuropeptide signal is aberrantly conveyed to neighboring ASG and ASJ sensory neurons via the tachykinin receptor TKR-1 expressed in these cells. Thus, AIY together with ASG and/or ASJ may constitute a neuropeptide-dependent modulatory pathway through which excessive NLP-58/TKR-1 signaling influences thermotaxis behavior. As a consequence, neural processing downstream of AIY may be altered, leading to abnormal thermotaxis behavior despite intact primary thermosensory input (Fig 7E). This model suggests that NLP-58 does not directly impair temperature sensing by itself but instead modulates thermotaxis through TKR-1-dependent signaling involving ASG or ASJ sensory neurons, thereby influencing temperature preference.

### Fezolinetant partially improves the thermotaxis behavior in *nlp-58*OE animals

We next examined whether fezolinetant influences the thermotaxis behavior of *nlp-58*OE animals. Fezolinetant is a selective antagonist of the neurokinin 3 receptor (NK3R) and represents the first non-hormonal therapeutic agent approved for the treatment of menopausal hot flashes. In mammals, this drug acts by antagonizing NKB–NK3R signaling [41]. *nlp-58*OE animals were treated with DMSO (0mM fezolinetant) or fezolinetant at concentrations of 0.05, 0.1, or 0.15 mM, respectively, for 1 hour before the thermotaxis assay.

Fezolinetant treatment shifted the distribution of *nlp-58*OE animals toward the cultivation-temperature region (Fig 8A). To quantify this effect, we measured the proportion of animals located in fractions 5–7(see Material and Methods). Paired estimation analysis revealed an increase in this proportion following fezolinetant treatment, with a significant increase at 0.15 mM (Fig 8B). Consistent with this result, analysis using the TTX index showed a significant improvement following treatment with 0.10 mM fezolinetant (S3 Fig). Together, these complementary measures indicate that fezolinetant partially improves the thermotaxis behavior of *nlp-58*OE animals.

**Fig 8:**
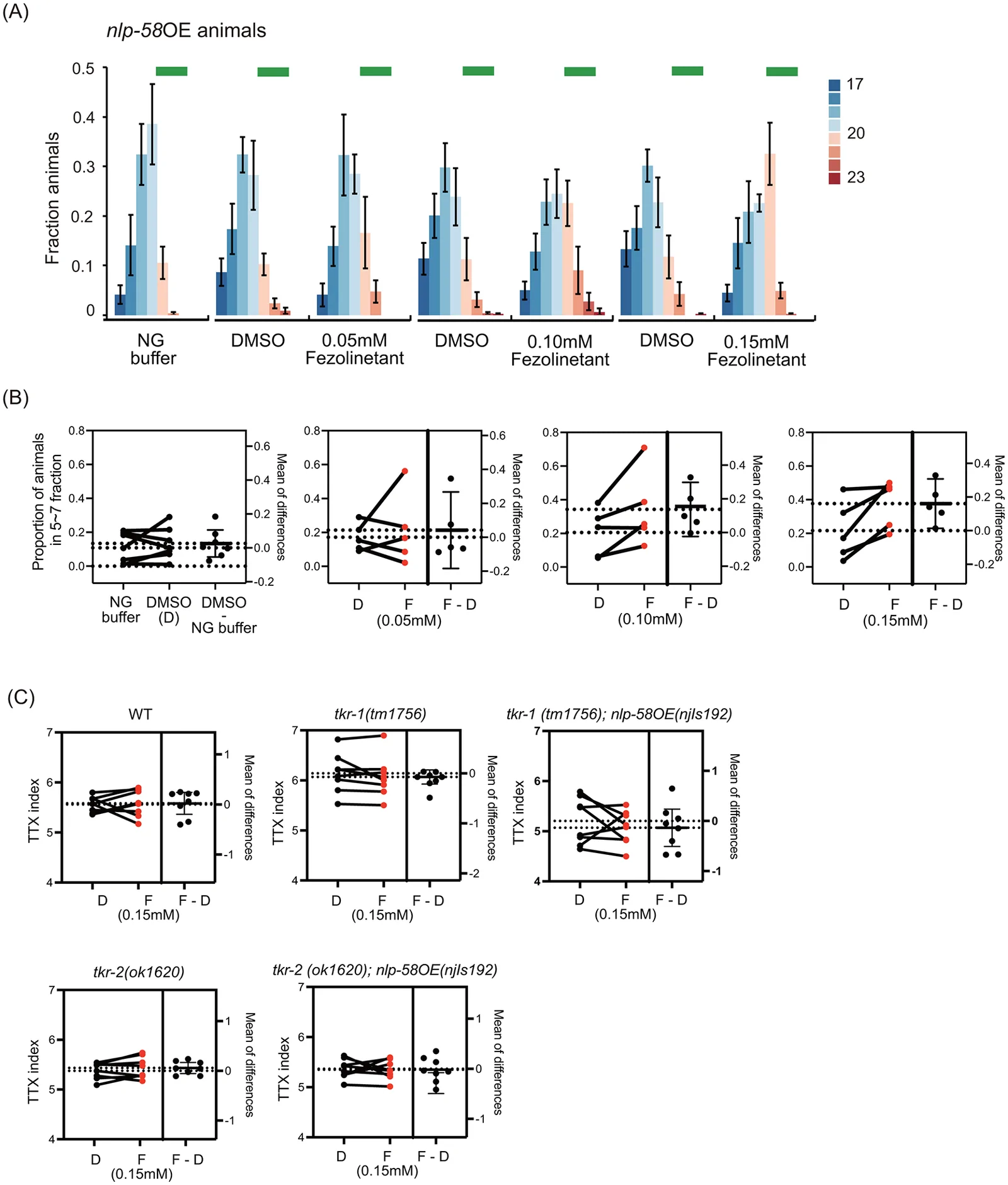
Fezolinetant partially improves abnormal thermotaxis behavior in *nlp-58*OE animals. (A) Distribution of *nlp-58*OE animals on the thermal gradient following treatment with DMSO or the NK3R antagonist fezolinetant. Animals cultivated at 23 °C were incubated with or without fezolinetant at final concentrations of 0.05, 0.1, or 0.15 mM for 1 h prior to the assay. Bars indicate the fraction of animals in each temperature region. The green-shaded region indicates fractions 5–7, corresponding to the region around the cultivation temperature; the proportion of animals within these fractions was quantified in (B). (B) Paired estimation plots showing the proportion of *nlp-58*OE animals located in fractions 5–7 following DMSO or fezolinetant treatment. Paired measurements from independent experiments are connected by lines. Mean paired differences (fezolinetant − DMSO) and their 95% confidence intervals are shown on the right. Treatment with 0.15 mM fezolinetant significantly increased the proportion of animals in fractions 5–7. NG buffer and DMSO controls are also shown. (C) Effects of 0.15 mM fezolinetant on thermotaxis in wild-type, *tkr-1(tm1756)*, *tkr-1(tm1756); nlp-58OE(njIs192)*, *tkr-2(ok1620)*, and *tkr-2(ok1620); nlp-58OE(njIs192)* animals. TTX indices for paired DMSO- and fezolinetant-treated groups are shown, with paired measurements connected by lines. Mean paired differences (fezolinetant − DMSO) and their 95% confidence intervals are shown on the right. Fezolinetant did not significantly alter the TTX index in these genetic backgrounds. D, DMSO; F, fezolinetant.

To determine whether the effect of fezolinetant depends on tachykinin receptor signaling, we examined its effect in wild-type, *tkr-1(tm1756), tkr-2(ok1620), nlp-58*OE*(njIs192); tkr-1(tm1756),* and *nlp-58*OE*(njIs192); tkr-2(ok1620)* animals. Fezolinetant did not significantly alter the TTX index in any of these genetic backgrounds (Fig 8C). Notably, the improvement observed in *nlp-58*OE animals was no longer detected in the *tkr-1; nlp-58*OE or *tkr-2; nlp-58*OE background. Together, these results indicate that fezolinetant preferentially modulates the thermotaxis defect associated with NLP-58 signaling rather than generally altering thermotaxis behavior and suggest that this effect may involve TKR-dependent signaling.

## Discussion

Hot flashes are among the most prevalent symptoms of menopause and substantially impair quality of life [4,5,42]. Recent studies have identified the tachykinin neuropeptide system, particularly neurokinin B (NKB) and its receptor NK3R, as a key contributor to menopausal vasomotor symptoms, and NK3R antagonists have shown clinical efficacy [8,9,12,43]. However, how altered tachykinin signaling modifies thermoregulatory neural circuits remains incompletely understood. In particular, distinguishing the effects of neuropeptide signaling on primary thermal sensation from those of other downstream processes and behavioral output remains an important challenge in complex mammalian systems.

In this study, we used C. elegans, in which thermosensory behavior can be analyzed at single-neuron resolution, to investigate how manipulation of tachykinin-like signaling modulates temperature-associated behavior. Temperature preference in C. elegans is regulated by a well-characterized circuit involving the AFD thermosensory neurons and downstream AIY interneurons [19,44]. We found that overexpression of *nlp-58*, which encodes a tachykinin-like neuropeptide related to mammalian NKB, showed thermotaxis behavioral defect without an apparent impairment of primary thermosensory responses. Cell-specific overexpression further showed that increased NLP-58 signaling from AIY is sufficient to reproduce the thermotaxis behavioral defect. Genetic analyses identified TKR-1 as the major tachykinin receptor contributing to this phenotype, and cell-specific rescue experiments demonstrated that TKR-1 expression in either ASG or ASJ sensory neurons is sufficient to restore the *nlp-58*OE phenotype in a *tkr-1* mutant background. Together, these findings support a model in which excessive NLP-58/TKR-1 signaling modulates thermotaxis behavior downstream or in parallel with the primary thermosensory pathway rather than directly disrupting temperature sensing.

Our finding further suggests that AIY participates in thermotaxis on multiple functional timescales. The AFD–AIY pathway mediates rapid responses to changes in environmental temperature [45]. In contrast, our results suggest that NLP-58 signaling from AIY may provide an additional modulatory pathway that influences thermotaxis circuit output. ASG and ASJ are classically defined as sensory neurons and are not central components of the canonical AFD–AIY thermotaxis pathway. Nevertheless, TKR-1 expression in either ASG or ASJ was sufficient to reproduce the abnormal thermotaxis phenotype of *nlp-58*OE animals in the *tkr-1* mutant background. ASJ has been reported to respond directly to temperature changes, suggesting that it possesses intrinsic thermosensory properties [46]. However, its specific contribution to thermotaxis behavior remains unclear. These observations are consistent with the possibility that ASG and ASJ participate in a modulatory pathway that influences thermotaxis outside the canonical AFD–AIY circuit. Because the present experiments demonstrate sufficiency but not necessity of TKR-1 function in these neurons, additional cell-specific loss-of-function experiments will be required to determine their endogenous contributions to NLP-58-dependent thermotaxis.

Our results also reveal a relationship between lipid metabolism and NLP-58 signaling. Loss of *dhs-4*, a gene involved in steroid hormone biosynthesis, altered thermotaxis behavior, and deletion of *nlp-58* suppressed the *dhs-4* mutant phenotype. Moreover, *nlp-58* expression was increased in *dhs-4* mutants as well as in animals lacking the nuclear hormone receptors genes *nhr-69*, *nhr-121*, and *nhr-14*. These genetic interactions suggest that DHS-4-dependent lipid metabolism and nuclear hormone receptor signaling can influence *nlp-58* expression and thermotaxis behavior. Importantly, however, exogenous β-estradiol treatment did not significantly change *nlp-58* expression in either wild-type or nuclear hormone receptor mutant backgrounds. Furthermore, the presence and physiological role of endogenous estrogen in *C. elegans* remain unresolved. Thus, our results do not establish estrogen as the endogenous signal linking DHS-4 or these nuclear hormone receptors to NLP-58. Instead, they support a broader model in which lipid metabolic and nuclear hormone receptor pathways modulate NLP-58 signaling, with the relevant endogenous ligand or metabolite remaining to be identified.

In addition to these regulatory mechanisms, our findings indicate that endogenous NLP-58/TKR-1 signaling contributes to age-dependent changes in thermotaxis behavior. Consistent with previous studies [32–34], the aged wild-type animals showed defect of the thermotaxis behavior. This age-dependent behavioral decline was significantly attenuated in *nlp-58(nj428)* and *tkr-1(tm1756)* mutants. In parallel, RNA-seq analysis revealed age-dependent increases in *tkr-1* and *tkr-2* transcript abundance, whereas *nlp-58* itself did not show a significant age-dependent change (Fig 3A). These observations suggest that changes in tachykinin receptor signaling, rather than increased *nlp-58* expression alone, may contribute to age-dependent modulation of thermotaxis.

The pharmacological experiments further support the involvement of TKR-1-dependent signaling in behavioral effects of NLP-58 overexpression. Fezolinetant, a selective antagonist of mammalian NK3R used clinically for menopausal vasomotor symptoms [47], partially improved the thermotaxis behavior of *nlp-58*OE animals, as supported by complementary analyses of animal distribution and the TTX index. Importantly, fezolinetant did not significantly alter thermotaxis in wild-type, *tkr-1*, or *tkr-2* mutant animals. Moreover, the positive shift in the TTX index observed following fezolinetant treatment of *nlp-58*OE animals was no longer apparent in the *tkr-1; nlp-58* OE background (Fig 8C). This finding is consistent with the pharmacological effect of fezolinetant depending, at least in part, on TKR-1 signaling. Previous biochemical studies have demonstrated that NLP-58-derived tachykinin-like peptides activate TKR-1 and TKR-2 [39,40]. However, whether fezolinetant directly interacts with or antagonizes TKR-1 has not been examined. Therefore, our results should not be interpreted as demonstrating that TKR-1 is a direct molecular target of fezolinetant. Direct pharmacological characterization of fezolinetant at TKR-1, for example using heterologous receptor assays, will be required to determine whether fezolinetant directly antagonized TKR-1.

Together, our findings identify the NLP-58/TKR-1 pathway as a modulatory component of the *C. elegans* thermotaxis circuit and reveal interactions among tachykinin-like neuropeptide signaling, lipid metabolic pathways, aging, and temperature preference. Additionally, we reveal a neuropeptide-dependent modulatory pathway in which NLP-58 signaling from AIY influences thermotaxis behavior through TKR-1 function in ASG and/or ASJ sensory neurons. Although mammalian thermoregulation and menopausal vasomotor symptoms involve physiological processes that are not recapitulated in *C. elegans*, the conservation of tachykinin signaling provides an opportunity to dissect fundamental principles of neuropeptide-mediated circuit modulation in a simple nervous system. Future studies combining cell-specific loss-of-function approaches with calcium imaging of ASG and ASJ during temperature stimulation in different *nlp-58* and *tkr-1* genetic backgrounds will be important for defining how NLP-58/TKR-1 signaling modulates thermosensory circuit activity. In parallel, direct pharmacological characterization of TKR-1 will be important for defining the endogenous circuit mechanism and determining the extent to which the pharmacological properties of mammalian NK3R signaling are conserved.

## Materials and methods

### *C. elegans* strains

All strains were maintained at 23°C on NGM agar plates seeded with *Escherichia coli* OP50 as a food source. The mutants, extrachromosomal arrays, and integrated transgenes used in this study are listed in S1 Table. We constructed strain IK4771, in which NLP-58 is overexpressed stably and continuously. Deletion of the second exon of *nlp-58* was generated using the CRISPR–Cas9 system as previously described, resulting in generating the *nj428* and *nj429* alleles [48].

### Transgene construction

To generate *nlp-58* overexpression, a 7,104 bp genomic fragment containing the *nlp-58* gene was amplified by PCR and microinjected into the wild-type N2 strain to generate extrachromosomal arrays [49]. The *njEx2134* arrays were subsequently integrated into the genome by gamma irradiation and backcrossed four times with the N2 strain [46].

To visualize *nlp-58* expression, a transcriptional reporter was constructed using PCR fusion [50]. Approximately 5 kb of the *nlp-58* promoter region, the entire genomic coding sequence, an *SL2::nls::GFP* fragment, and the *nlp-58* 3′ untranslated region (3′ UTR) were amplified and fused by PCR. For *tkr-1* expression analysis, a 3.4 kb fragment upstream of the first exon of *tkr-1* was cloned into pPD95.75 vector with TagRFP substituted for *gfp* (pMJ085). pPD95.75 was a gift from Andrew Fire (Addgene plasmid # 1494; http://n2t.net/addgene:1494; RRID:Addgene1494).

To visualize neuronal subtypes, an 884 bp fragment upstream of the *ttx-3* start codon (AIY-specific) was cloned into pMJ085. For cell-specific *nlp-58* overexpression, a 285 bp *nlp-58* cDNA fragment was inserted into pPD95.75 under the control of 3.8 kb *nlp-58*, 0.8 kb *ttx-3*, 1.6 kb *gcy- 21*, or 0.8 kb *dat-1* promoters, driving expression in endogenous *nlp-58*–expressing neurons, AIY interneurons, or dopaminergic neurons (CEP and ADE), respectively.

For *tkr-1* functional rescue, the GFP coding sequence in pSN874 (*srh-11p::GFP*, ASJ-specific) or pSN891 (*gcy-21p::GFP*, ASG-specific) plasmids was replaced with a 1,221 bp *tkr-1* cDNA. These constructs were used to restore *tkr-1* expression selectively in ASG, ASJ, or both neurons.

### Measurement of body size

Body length was measured from bright-field images of synchronized young adult animals using ImageJ (National Institutes of Health).

### Egg-laying assay

Synchronized eggs were obtained by bleaching gravid adults as described previously [51] and approximately 100 eggs cultivated at 23°C on NGM agar medium. After two days, L4-stage animals were individually transferred onto 3.5 cm NGM plates (one worm per plate). After 20 hours, adults were removed and the plates were further incubated until progeny reached adulthood. To immobilize worms for counting, chloroform vapor was applied to the lid, and the plates were immediately sealed. The total number of progeny was then counted, confirming that all eggs hatched successfully in each assay.

### Defecation cycle assay

Synchronized worms were obtained by bleaching as described previously [51]. Approximately 100 eggs were cultured at 23°C on NGM agar medium. After two days, L4-stage animals were individually transferred onto 3.5 cm NGM plates (one worm per plate). After 6 hours, these were considered Day 1 adults. The defecation cycle was measured under a stereomicroscope by recording the interval between successive defecation events. Measurements were performed using adult animals from Day 1 to Day 5.

### Measurement of developmental speed

Synchronized L1 worms were obtained by standard bleaching [51] and allowed to develop on NGM plates seeded with *Escherichia coli* OP50 at 19.6°C. After 52 h, animals were classified according to their developmental stage (L4, young adult, or adult) based on vulval morphology under a stereomicroscope. The percentage of animals at each developmental stage was calculated for each genotype.

### Chemotaxis behavioral assay

Chemotaxis assays were performed essentially as described previously [26,52]. Animals were conditioned for 4 hours on NGM plates containing 25 mM NaCl and seeded with *E. coli* OP50. After conditioning, animals were collected, washed, and placed at the center of assay plates containing NaCl concentration gradients. Animals were allowed to migrate for 60 min, after which the numbers of animals that migrated toward the high- and low-NaCl regions or remained at the starting region were counted. The chemotaxis index was calculated as:

Chemotaxis index = (*N*high – *N*low)/ (*N*total – *N*start), where *N*high and *N*low represent the numbers of animals in the high- and low-NaCl regions, respectively, *N*total represents the total number of animals scored, and *N*start represents the number of animals remaining at the starting region.

### Thermotaxis behavioral assay

Thermotaxis assays were performed as previously described [53,54]. Adult animals were cultivated on NGM plates at 23°C for three days. Animals were collected in NG buffer (0.3% NaCl, 1 mM MgSO_4_, 1 mM CaCl_2_, 25 mM KPO_4_, pH 6.0) and washed twice with the same buffer. Approximately 100–150 animals were placed at the center of a thermotaxis assay plate with a linear temperature gradient (17–23°C; 0.45–0.5°C/cm). Animals were allowed to move freely for 1 hour, after which locomotion was stopped using chloroform vapor. Each plate was divided into eight equal fractions, and the number of animals in each fraction was counted to quantify thermotaxis behavior.

Thermotaxis behavior was quantified using a thermotaxis index (TTX index). Each fraction was assigned a score from 1 (coldest region) to 8 (warmest region), and the TTX index was calculated as the weighted average of animal distribution across the gradient using the following formula: TTX index = Σ(Si × Ni)/N, where Si represents the score of each fraction, Ni represents the number of animals in each fraction, and N represents the total number of animals scored. Differences in thermotaxis behavior between genotypes were evaluated by comparing TTX indices using the statistical tests indicated in each figure legend. Throughout this study, “abnormal thermotaxis” refers to a statistically significant difference in TTX index relative to wild-type animals, whereas “behavioral suppression” or “behavioral rescue” refers to a statistically significant improvement relative to the *nlp-58*OE animals.

### Preparation of aged animals

Age-synchronized animals were prepared by allowing 15–20 gravid hermaphrodites to lay eggs on NGM plates seeded with E. coli OP50 for 2 h at 23°C, after which the adult animals were removed. Three days later, the resulting adult progeny were designated as day 1 (D1) adults. D1 adults were collected from the plates using NG buffer. Animals were transferred to glass tubes and washed twice with NG buffer to separate adults from eggs and larvae, taking advantage of the faster sedimentation of adults. The collected adults were then transferred to fresh NGM plates seeded with OP50 and maintained at 23°C. From D2 to D4 of adulthood, adult animals were transferred daily to fresh OP50-seeded NGM plates after collection and three washes with NG buffer to remove progeny. On D5 of adulthood, animals were collected and used for thermotaxis assays.

### RNA sequencing and gene expression analysis

Synchronization of the animals was performed by collecting young adult animals without embryos in their gonads and transferring them onto new plates. Animals were then transferred to new plates every two days and collected on day 1, day 3, day 5, and day 9 of adulthood. 100 animals were collected on each respective day and total RNA from age-synchronized animals was extracted using QIAzol Lysis Reagent (Qiagen, Germany). Samples were then sent to the Bioinformatics Center, NGS Core Facility at Osaka University for quantification, library preparation, and sequencing using the Agilent RNA 6000 Nano Kit (Agilent Technologies, Santa Clara), TruSeq Stranded mRNA Library Prep Kit (Illumina, San Diego), and the Illumina NovaSeq 6000 (Illumina, San Diego), respectively.

After sequencing, raw data quality was first assessed using FASTQC (version 0.11.9) [55] before adapters were trimmed off of the reads by using Cutadapt (version 4.6) [56]. Cleaned reads were then mapped to the *C. elegans* reference genome (database version WBPS19, annotation version WS285) using Salmon (version 1.10.2) [57]. Transcripts were compared against a decoy-aware transcriptome and quantified using Salmon quant to receive lengthScaledTPM values. Data was then imported using tximport (version 1.37.5) [58] and replicates were merged before being compared using Tukey’s Multiple Comparison Test to create the gene expression graphs. Although data from day 1 animals were examined, it was excluded from the analysis due to the high variation caused by the remaining embryos in the animals’ bodies.

### Calcium imaging in AFD neurons

Calcium imaging of temperature-dependent AFD neuronal activity using GCaMP3 and TagRFP fluorescence was performed as described previously [53]. A linear thermal stimulus ranging from 17 °C to 25 °C was applied, and calcium responses were recorded. For quantitative analysis, the average calcium response during the 100–120 s and 140–160 s interval of the warming phase was calculated and compared between wild-type and *nlp-58* overexpression (OE) animals.

### Identification of *nlp-58*-expression cells

The multi-color images were acquired using a spinning-disk confocal 3D microscope system (OSB4E) following a modified protocol [59]. Animals expressing genomic *nlp-58::sl2::gfp* driven by endogenous promoter were cultivated at 23°C for 3 days. Prior to imaging, young adult animals were picked and washed with NGM buffer. Animals were then loaded into an olfactory chip as described previously [60] and immobilized with 50 mM sodium azide in NGM buffer. Image preprocessing and reconstruction were performed using a custom Python-based pipeline and Fiji [61]. Cell identity was determined either manually or by following the procedures described in the NeuroPAL manual (available at https://www.hobertlab.org/neuropal/) and the methods of Yemini [35], and was further confirmed by co-localization with the AIY-specific fluorescent markers TagRFP.

### Comparison of fluorescent *nlp-58* expression

To compare *nlp-58* expression levels at the transcriptional level among different strains, we used animals expressing GFP under the induction of the endogenous *nlp-58* promoter, together with TagRFP expressed specifically in AIY interneurons as an internal reference. This dual-fluorescence strategy enabled normalization of *nlp-58* expression to AIY neuron identity and morphology.

Fluorescence images were acquired using a KEYENCE BZ-X810 fluorescence microscope equipped with GFP and RFP filter sets, a 40x objective lens, and an exposure time of 5 s for all samples. For each animal, z-stack images covering approximately 40 μm were collected at 1 μm intervals. Image processing and quantitative analysis were performed using Fiji [61]. *nlp-58* expression levels were quantified by calculating the fluorescence intensity ratio of GFP to TagRFP within the AIY neuron. To examine the temperature dependence of *nlp-58* expression, the reporter strain *oskEx36197* was cultivated either at 23 °C or at 15 °C. Fluorescence quantification was performed using the same imaging and analysis procedures described above.

### DiO staining

To visualize amphid sensory neurons (ASK, ADL, ASI, ASH, AWB, and ASJ), DiO staining was performed as previously described [62]. The *tkr-1p::TagRFP* expressing transgenic animals were collected from NGM plates and washed twice with M9 buffer, then incubated in 1 mL of M9 buffer containing 10 µg/mL DiO (Molecular Probes) while rotating at room temperature for 1 hour. After washing with fresh M9 buffer, animals were transferred to NGM plates and allowed to recover for 2 hours. Stained animals were immobilized with sodium azide on agar pads and observed under a fluorescence microscope.

### Fezolinetant treatment

To examine whether fezolinetant could rescue the thermotaxis defects of *nlp-58*OE animals, worms cultivated at 23 °C for 3 days were collected using NG buffer and transferred to 1.5-mL Eppendorf tubes. Fezolinetant (MedChemExpress Cat. no. HY-19632) dissolved in DMSO was mixed with OP50 and added to the tubes to yield final concentrations of 0.05, 0.1, and 0.15 mM in a total volume of 1 ml. As a control, an equivalent amount of DMSO was added to OP50-containing NG buffer. Animals were incubated in the fezolinetant-containing or control solutions supplemented with OP50 for approximately 1 hour at 23°C with gentle rotation. After that, tubes were left undisturbed to allow worms to settle by gravity, and the supernatant was removed. Worms were washed once with NG buffer and immediately subjected to the thermotaxis assay on a linear temperature gradient plate.

### Statistics

Statistical analyses were performed using GraphPad Prism 10 (GraphPad Software, San Diego, CA, USA). Data was analyzed using unpaired two-tailed Student’s t-test or one-way ANOVA followed by Dunnett’s or Tukey’s multiple comparison tests as appropriate. Details of statistical tests, sample sizes, and exact p-values are provided in the figure legends.

## Supporting information

S1 Fig Phenotypic characterization of *nlp-58*OE animals. (A) Representative stereomicroscope images of day1 adult wild-type (N2) and *nlp-58*OE (IK4771) animals. (B) Body length of day 1 adult wild-type and *nlp-58*OE animals. The estimation plot shows individual measurements (left) and the mean difference between nlp-58OE and wild-type animals with the 95% confidence interval (right). (C) Number of eggs laid by individual animals during a 20-hour period at the L4-adult transition stage. Each dot represents one animal; lines indicate mean. *** p < 0.0001 by unpaired two-tailed Student’s t-test. (D) Defecation cycle analysis of synchronized animals cultivated from eggs to adulthood. Day 1 corresponds to the first day of adulthood, and measurements were performed up to Day 4. The defecation cycle was defined as the interval between consecutive expulsion events. In WT animals, the cycle length gradually increased with aging. A slight tendency for increased variability in defecation cycle length was seen in Day 3–Day 4 *nlp-58*OE adults. *** p < 0.0001, all other comparisons were not significant by Tukey’s multiple comparison test. (E) Developmental stage distribution of wild-type and *nlp-58*OE animals at 52h after synchronization. Animals were classified as L4, young adult, or adult. (F) Locomotion speed of wild-type and *nlp-58*OE animals. The estimation plot shows individual measurements (left) and the mean difference between *nlp-58*OE and wild-type animals with the 95% confidence interval (right). (G) Salt chemotaxis behavior of wild-type and *nlp-58*OE animals after conditioning with food at 25 mM NaCl for 4 h. The schematic illustrates the conditioning and chemotaxis assay. The chemotaxis index (CI) was calculated as (Nhigh − Nlow)/(Ntotal − Nstart), where Nhigh and Nlow are the numbers of animals in the high- and low-NaCl regions, respectively, Ntotal is the total number of animals scored, and Nstart is the number of animals remaining at the starting region. No significant difference in the chemotaxis index was detected between wild-type and *nlp-58*OE animals.
(EPS)

S2 Fig Comparison of *nlp-58* expression at different cultivation temperature. (A) Representative fluorescence images of animals expressing *nlp-58::GFP* together with an AIY-specific TagRFP marker. The dotted line indicates the AIY interneurons. (B) Comparison of NLP-58 expression in AIY neurons according to cultivation temperatures. KDK36197 animals cultivated at 15 °C exhibited increased *nlp-58::GFP* fluorescence in AIY compared with those cultured at 23 °C, indicating that *nlp-58* expression in AIY is temperature dependent. *** p < 0.0001 by unpaired two-tailed Student’s t-test.
(EPS)

S3 Fig Effects of fezolinetant on the TTX index of *nlp-58*OE animals. Paired estimation plots of the TTX index following treatment with DMSO or fezolinetant at the indicated concentrations. Paired measurements from independent experiments are connected by lines. Mean paired differences (fezolinetant − DMSO) and their 95% confidence intervals are shown on the right. Treatment with 0.10 mM fezolinetant significantly increased the TTX index compared with the paired DMSO control. Comparisons between NG buffer and DMSO are also shown. D, DMSO; F, fezolinetant.
(EPS)

S1 Table Raw data and strains used in this study. (XLSX)

S1 File Confirmation of authorship change. (PDF)
