# Supplementary material for: Tachykinin-like NLP-58 signaling through TKR-1 modulates thermotaxis behavior in Caenorhabditis elegans
Source: PLoS Genet. 2026 Nov 11;22(11):e1012346. doi: 10.1371/journal.pgen.1012346 (PMC13754146; doi:10.1371/journal.pgen.1012346)
Supplement: Supplementary file 1 [file pgen.1012346.s005.pdf]

差出人: matsuyam@pu-toyama.ac.jp
件名: Re: Confirmation of authorship change for PLOS Genetics manuscript PGENETICS-D-26-00144R1
日付: 2026年9月22日 9:24
宛先: nishiwaki@kwansei.ac.jp
CC: gr0609hs@ed.ritsumei.ac.jp、 msjang@nsc.nagoya-cu.ac.jp、 ryoga.suzuki.lab@gmail.com、 yukimasashibata@cibr.ac.cn 、 zhangxiaolin@cibr.ac.cn、 kokimura@nsc.nagoya-cu.ac.jp、 yukubota@fc.ritsumei.ac.jp、 maito@sk.ritsumei.ac.jp、 ikuemori@cibr.ac.cn

---

Dear Dr. Moon Sun Jang,

I confirm that I agree with the addition of Xiaolin Zhang as an author of manuscript PGENETICS-D-26-00144R1.

Best,
Hironori J. Matsuyama

--
Hironori J. Matsuyama, Ph.D.
Assistant Professor
Laboratory of Systems Science,
Department of Data Science,
Faculty of Information Engineering,
Toyama Prefectural University, Imizu, Japan.
Email: matsuyam@pu-toyama.ac.jp
Phone: 0766-56-7500 (ext. 3105)

> 2026/09/22 9:14、nishiwaki@kwansei.ac.jpのメール:
>
> Dear Dr. Moon Sun Jang,
>
> I confirm that I agree with the addition of Xiaolin Zhang as an author of manuscript PGENETICS-D-26-00144R1.
>
> Best,
> Kiyoji Nishiwaki
>
> Get Outlook for Android
>
> ---
>
> **From:** msjang@nsc.nagoya-cu.ac.jp <msjang@nsc.nagoya-cu.ac.jp>
> **Sent:** Monday, 21 September 2026 21:16:21
> **To:** matsuyam@pu-toyama.ac.jp <matsuyam@pu-toyama.ac.jp>; ryoga.suzuki.lab@gmail.com <ryoga.suzuki.lab@gmail.com>; ＬＡＷ Ｋｉｎｇ Ｃｈｕｅｎ (gr0609hs) <gr0609hs@ed.ritsumei.ac.jp>; yukimasashibata@cibr.ac.cn <yukimasashibata@cibr.ac.cn>;zhangxiaolin@cibr.ac.cn <zhangxiaolin@cibr.ac.cn>; kokimura@nsc.nagoya-cu.ac.jp <kokimura@nsc.nagoya-cu.ac.jp>; 久保田 幸彦 (yukubota) <yukubota@fc.ritsumei.ac.jp>; 伊藤 將弘(maito) <maito@sk.ritsumei.ac.jp>; nishiwaki@kwansei.ac.jp<nishiwaki@kwansei.ac.jp>; ikuemori@cibr.ac.cn <ikuemori@cibr.ac.cn>
> **Cc:** msjang@nsc.nagoya-cu.ac.jp <msjang@nsc.nagoya-cu.ac.jp>
> **Subject:** Confirmation of authorship change for PLOS Genetics manuscript PGENETICS-D-26-00144R1
>
> Dear all,
>
> PLOS Genetics has requested written confirmation from all authors regarding the authorship change in our revised manuscript entitled "Tachykinin-like NLP-58 signaling through TKR-1 modulates thermotaxis behavior in Caenorhabditis elegans" (Manuscript ID: PGENETICS-D-26-00144R1).
>
> Xiaolin Zhang has been added as an author based on her contribution to the aged-worm thermotaxis experiments included in the revised manuscript.

If you agree with this authorship change, could you please reply to this email with the following statement:

“I confirm that I agree with the addition of Xiaolin Zhang as an author of manuscript PGENETICS-D-26-00144R1.”

Thank you very much for your cooperation.

===============
共著者の皆様

PLOS Geneticsより、改訂に伴う著者変更について、追加著者を含む全著者から書面による同意確認を提出するよう連絡がありました。

今回の改訂では、加齢線虫を用いた温度走性実験への貢献に基づき、Xiaolin Zhangさんを著者として追加しています。

この著者追加にご同意いただける場合は、本メールへの返信として以下の英文をそのままお送りいただけますでしょうか。

“I confirm that I agree with the addition of Xiaolin Zhang as an author of manuscript PGENETICS-D-26-00144R1.”

PLOSへの提出資料として使用させていただきます。

お忙しいところ恐れ入りますが、よろしくお願いいたします。

Best regards,
Moon Sun Jang

差出人: **Ryoga Suzuki** ryoga.suzuki.lab@gmail.com
件名: Re: Confirmation of authorship change for PLOS Genetics manuscript PGENETICS-D-26-00144R1
日付: 2026年9月22日 15:09
宛先: msjang@nsc.nagoya-cu.ac.jp
CC: matsuyam@pu-toyama.ac.jp、 gr0609hs@ed.ritsumei.ac.jp、 yukimasashibata@cibr.ac.cn、 zhangxiaolin@cibr.ac.cn、 kokimura@nsc.nagoya-cu.ac.jp、 yukubota@fc.ritsumei.ac.jp、 maito@sk.ritsumei.ac.jp、 nishiwaki@kwansei.ac.jp、 ikuemori@cibr.ac.cn

---

Dear Dr. Moon Sun Jang

I confirm that I agree with the addition of Xiaolin Zhang as an author of manuscript PGENETICS-D-26-00144R1.
Best,
Suzuki Ryoga

2026年9月21日(月) 21:16 <msjang@nsc.nagoya-cu.ac.jp>:

> Dear all,
>
> PLOS Genetics has requested written confirmation from all authors regarding the authorship change in our revised manuscript entitled “Tachykinin-like NLP-58 signaling through TKR-1 modulates thermotaxis behavior in Caenorhabditis elegans” (Manuscript ID: PGENETICS-D-26-00144R1).
>
> Xiaolin Zhang has been added as an author based on her contribution to the aged-worm thermotaxis experiments included in the revised manuscript.
>
> If you agree with this authorship change, could you please reply to this email with the following statement:
>
> “I confirm that I agree with the addition of Xiaolin Zhang as an author of manuscript PGENETICS-D-26-00144R1.”
>
> Thank you very much for your cooperation.
>
> ==============
> 共著者の皆様
>
> PLOS Geneticsより、改訂に伴う著者変更について、追加著者を含む全著者から書面による同意確認を提出するよう連絡がありました。
>
> 今回の改訂では、加齢線虫を用いた温度走性実験への貢献に基づき、Xiaolin Zhangさんを著者として追加しています。
>
> この著者追加にご同意いただける場合は、本メールへの返信として以下の英文をそのままお送りいただけますでしょうか。
>
> “I confirm that I agree with the addition of Xiaolin Zhang as an author of manuscript PGENETICS-D-26-00144R1.”
>
> PLOSへの提出資料として使用させていただきます。
>
> お忙しいところ恐れ入りますが、よろしくお願いいたします。
>
> Best regards,
> Moon Sun Jang

差出人: **LAW King Chuen** gr0609hs@ed.ritsumei.ac.jp
件名: Re: Confirmation of authorship change for PLOS Genetics manuscript PGENETICS-D-26-00144R1
日付: 2026年9月22日 6:51
宛先: msjang@nsc.nagoya-cu.ac.jp、 matsuyam@pu-toyama.ac.jp、 ryoga.suzuki.lab@gmail.com、 yukimasashibata@cibr.ac.cn、 zhangxiaolin@cibr.ac.cn、 kokimura@nsc.nagoya-cu.ac.jp、 KUBOTA YUKIHIKO yukubota@fc.ritsumei.ac.jp、 ITO MASAHIRO maito@sk.ritsumei.ac.jp、 ikuemori@cibr.ac.cn
CC: msjang@nsc.nagoya-cu.ac.jp、 nishiwaki@kwansei.ac.jp

LC

Dear Dr. Moon Sun Jang,

I confirm that I agree with the addition of Xiaolin Zhang as an author of manuscript PGENETICS-D-26-00144R1.

Best,

King

Get [Outlook for Android]

From: msjang@nsc.nagoya-cu.ac.jp <msjang@nsc.nagoya-cu.ac.jp>
Sent: Monday, 21 September 2026 21:16:21
To: matsuyam@pu-toyama.ac.jp <matsuyam@pu-toyama.ac.jp>; ryoga.suzuki.lab@gmail.com <ryoga.suzuki.lab@gmail.com>; ＬＡＷ Ｋｉｎｇ Ｃｈｕｅｎ (gr0609hs) <gr0609hs@ed.ritsumei.ac.jp>; yukimasashibata@cibr.ac.cn <yukimasashibata@cibr.ac.cn>; zhangxiaolin@cibr.ac.cn <zhangxiaolin@cibr.ac.cn>; kokimura@nsc.nagoya-cu.ac.jp <kokimura@nsc.nagoya-cu.ac.jp>; 久保田 幸彦(yukubota) <yukubota@fc.ritsumei.ac.jp>; 伊藤 將弘(maito) <maito@sk.ritsumei.ac.jp>; nishiwaki@kwansei.ac.jp <nishiwaki@kwansei.ac.jp>; ikuemori@cibr.ac.cn <ikuemori@cibr.ac.cn>
Cc: msjang@nsc.nagoya-cu.ac.jp <msjang@nsc.nagoya-cu.ac.jp>
Subject: Confirmation of authorship change for PLOS Genetics manuscript PGENETICS-D-26-00144R1

Dear all,

PLOS Genetics has requested written confirmation from all authors regarding the authorship change in our revised manuscript entitled " Tachykinin-like NLP-58 signaling through TKR-1 modulates thermotaxis behavior in Caenorhabditis elegans" (Manuscript ID: PGENETICS-D-26-00144R1).

Xiaolin Zhang has been added as an author based on her contribution to the aged-worm thermotaxis experiments included in the revised manuscript.

If you agree with this authorship change, could you please reply to this email with the following statement:

"I confirm that I agree with the addition of Xiaolin Zhang as an author of manuscript PGENETICS-D-26-00144R1."

Thank you very much for your cooperation.

==============
共著者の皆様

PLOS Geneticsより、改訂に伴う著者変更について、追加著者を含む全著者から
書面による同意確認を提出するよう連絡がありました。

書面による同意確認を提出するよう連絡がありました。

今回の改訂では、加齢線虫を用いた温度走性実験への貢献に基づき、Xiaolin Zhangさんを著者として追加しています。

この著者追加にご同意いただける場合は、本メールへの返信として以下の英文をそのままお送りいただけますでしょうか。

“I confirm that I agree with the addition of Xiaolin Zhang as an author of manuscript PGENETICS-D-26-00144R1.”

PLOSへの提出資料として使用させていただきます。

お忙しいところ恐れ入りますが、よろしくお願いいたします。

Best regards,
Moon Sun Jang

差出人: **Yukimasa Shibata** yukimasashibata@cibr.ac.cn
件名: Re: Confirmation of authorship change for PLOS Genetics manuscript PGENETICS-D-26-00144R1
日付: 2026年9月21日 22:36
宛先: msjang@nsc.nagoya-cu.ac.jp
CC: matsuyam@pu-toyama.ac.jp、 ryoga.suzuki.lab@gmail.com、 gr0609hs@ed.ritsumei.ac.jp、 zhangxiaolin@cibr.ac.cn、 kokimura@nsc.nagoya-cu.ac.jp、 yukubota@fc.ritsumei.ac.jp、 maito@sk.ritsumei.ac.jp、 nishiwaki@kwansei.ac.jp、 ikuemori@cibr.ac.cn

YS

Dear Dr, Moon Sun Jang

I confirm that I agree with the addition of Xiaolin Zhang as an author
of manuscript PGENETICS-D-26-00144R1.

Best,

Yukimasa Shibata

> -----Original Messages-----
> From: msjang@nsc.nagoya-cu.ac.jp
> Send time:Monday, 21/09/2026 20:16:21
> To: matsuyam@pu-toyama.ac.jp, ryoga.suzuki.lab@gmail.com, gr0609hs@ed.ritsumei.ac.jp, yukimasashibata@cibr.ac.cn, zhangxiaolin@cibr.ac.cn, kokimura@nsc.nagoya-cu.ac.jp, yukubota@fc.ritsumei.ac.jp, maito@sk.ritsumei.ac.jp, nishiwaki@kwansei.ac.jp, ikuemori@cibr.ac.cn
> Cc: msjang@nsc.nagoya-cu.ac.jp
> Subject: Confirmation of authorship change for PLOS Genetics manuscript PGENETICS-D-26-00144R1
>
> Dear all,
>
> PLOS Genetics has requested written confirmation from all authors regarding the authorship change in our revised manuscript entitled "Tachykinin-like NLP-58 signaling through TKR-1 modulates thermotaxis behavior in Caenorhabditis elegans" (Manuscript ID: PGENETICS-D-26-00144R1).
>
> Xiaolin Zhang has been added as an author based on her contribution to the aged-worm thermotaxis experiments included in the revised manuscript.
>
> If you agree with this authorship change, could you please reply to this email with the following statement:
>
> "I confirm that I agree with the addition of Xiaolin Zhang as an author of manuscript PGENETICS-D-26-00144R1."
>
> Thank you very much for your cooperation.
>
> ==============
> 共著者の皆様
>
> PLOS Geneticsより、改訂に伴う著者変更について、追加著者を含む全著者から書面による同意確認を提出するよう連絡がありました。
>
> 今回の改訂では、加齢線虫を用いた温度走性実験への貢献に基づき、Xiaolin Zhangさんを著者として追加しています。
>
> この著者追加にご同意いただける場合は、本メールへの返信として以下の英文をそのままお送りいただけますでしょうか。
>
> "I confirm that I agree with the addition of Xiaolin Zhang as an author of manuscript PGENETICS-D-26-00144R1."
>
> PLOSへの提出資料として使用させていただきます。
>
> お忙しいところ恐れ入りますが、よろしくお願いいたします。
>
> Best regards,
> Moon Sun Jang

差出人: **张晓琳** zhangxiaolin@cibr.ac.cn
件名: Re: Confirmation of authorship change for PLOS Genetics manuscript PGENETICS-D-26-00144R1
日付: 2026年9月21日 22:26
宛先: msjang@nsc.nagoya-cu.ac.jp

张

Dear Moon Sun,

I hope this message finds you well.
I confirm that I agree with the addition of Xiaolin Zhang as an author of manuscript PGENETICS-D-26-00144R1.

Thanks very much!

Best,
Xiaolin

> -----原始郵件-----
> 發件人: msjang@nsc.nagoya-cu.ac.jp
> 發送時間:2026-09-21 20:16:21 (星期一)
> 收件人: matsuyam@pu-toyama.ac.jp, ryoga.suzuki.lab@gmail.com, gr0609hs@ed.ritsumei.ac.jp, yukimasashibata@cibr.ac.cn, zhangxiaolin@cibr.ac.cn, kokimura@nsc.nagoya-cu.ac.jp, yukubota@fc.ritsumei.ac.jp, maito@sk.ritsumei.ac.jp, nishiwaki@kwansei.ac.jp, ikuemori@cibr.ac.cn
> 抄送: msjang@nsc.nagoya-cu.ac.jp
> 主題: Confirmation of authorship change for PLOS Genetics manuscript PGENETICS-D-26-00144R1
>
> Dear all,
>
> PLOS Genetics has requested written confirmation from all authors regarding the authorship change in our revised manuscript entitled “Tachykinin-like NLP-58 signaling through TKR-1 modulates thermotaxis behavior in Caenorhabditis elegans” (Manuscript ID: PGENETICS-D-26-00144R1).
>
> Xiaolin Zhang has been added as an author based on her contribution to the aged-worm thermotaxis experiments included in the revised manuscript.
>
> If you agree with this authorship change, could you please reply to this email with the following statement:
>
> “I confirm that I agree with the addition of Xiaolin Zhang as an author of manuscript PGENETICS-D-26-00144R1.”
>
> Thank you very much for your cooperation.
>
> ===============
> 共著者の皆様
>
> PLOS Geneticsより、改訂に伴う著者変更について、追加著者を含む全著者から
> 書面による同意確認を提出するよう連絡がありました。
>
> 今回の改訂では、加齢線虫を用いた温度走性実験への貢献に基づき、Xiaolin Zhangさんを著者として追加しています。
>
> この著者追加にご同意いただける場合は、本メールへの返信として以下の英文を
> そのままお送りいただけますでしょうか。
>
> “I confirm that I agree with the addition of Xiaolin Zhang as an author of manuscript PGENETICS-D-26-00144R1.”
>
> PLOSへの提出資料として使用させていただきます。
>
> お忙しいところ恐れ入りますが、よろしくお願いいたします。
>
> Best regards,
> Moon Sun Jang

差出人: **Kimura, Kotaro** kokimura@nsc.nagoya-cu.ac.jp
件名: Re: Confirmation of authorship change for PLOS Genetics manuscript PGENETICS-D-26-00144R1
日付: 2026年9月21日 22:27
宛先: Moon Sun Jang msjang@nsc.nagoya-cu.ac.jp

KK

Dear Jang san,

I confirm that I agree with the addition of Xiaolin Zhang as an author of manuscript PGENETICS-D-26-00144R1.

Best regards,

Kotaro Kimura

> 2026/09/21 午後9:16、msjang@nsc.nagoya-cu.ac.jpのメール:
>
> Dear all,
>
> PLOS Genetics has requested written confirmation from all authors regarding the authorship change in our revised manuscript entitled “ Tachykinin-like NLP-58 signaling through TKR-1 modulates thermotaxis behavior in Caenorhabditis elegans” (Manuscript ID: PGENETICS-D-26-00144R1).
>
> Xiaolin Zhang has been added as an author based on her contribution to the aged-worm thermotaxis experiments included in the revised manuscript.
>
> If you agree with this authorship change, could you please reply to this email with the following statement:
>
> “I confirm that I agree with the addition of Xiaolin Zhang as an author of manuscript PGENETICS-D-26-00144R1.”
>
> Thank you very much for your cooperation.
>
> ==============
> 共著者の皆様
>
> PLOS Geneticsより、改訂に伴う著者変更について、追加著者を含む全著者から書面による同意確認を提出するよう連絡がありました。
>
> 今回の改訂では、加齢線虫を用いた温度走性実験への貢献に基づき、Xiaolin Zhangさんを著者として追加しています。
>
> この著者追加にご同意いただける場合は、本メールへの返信として以下の英文をそのままお送りいただけますでしょうか。
>
> “I confirm that I agree with the addition of Xiaolin Zhang as an author of manuscript PGENETICS-D-26-00144R1.”
>
> PLOSへの提出資料として使用させていただきます。
>
> お忙しいところ恐れ入りますが、よろしくお願いいたします。
>
> Best regards,
> Moon Sun Jang

差出人: **KUBOTA YUKIHIKO** yukubota@fc.ritsumei.ac.jp
件名: Re: Confirmation of authorship change for PLOS Genetics manuscript PGENETICS-D-26-00144R1
日付: 2026年9月22日 10:57
宛先: Moon Sun Jang msjang@nsc.nagoya-cu.ac.jp
CC: matsuyam@pu-toyama.ac.jp、 LAW King Chuen gr0609hs@ed.ritsumei.ac.jp、 nishiwaki@kwansei.ac.jp、 ryoga.suzuki.lab@gmail.com、 yukimasashibata@cibr.ac.cn、 zhangxiaolin@cibr.ac.cn、 kokimura@nsc.nagoya-cu.ac.jp、 ITO MASAHIRO maito@sk.ritsumei.ac.jp、 ikuemori@cibr.ac.cn

KY

Dear Dr. Moon Sun Jang,

I confirm and agree with the addition of Xiaolin Zhang as an author of our manuscript "PGENETICS-D-26-00144R1".

Best,
Yukihiko Kubota

差出人: matsuyam@pu-toyama.ac.jp <matsuyam@pu-toyama.ac.jp>
送信日時: 2026年9月22日 9:24
宛先: nishiwaki@kwansei.ac.jp <nishiwaki@kwansei.ac.jp>
CC: ＬＡＷ Ｋｉｎｇ Ｃｈｕｅｎ(gr0609hs) <gr0609hs@ed.ritsumei.ac.jp>; msjang@nsc.nagoya-cu.ac.jp <msjang@nsc.nagoya-cu.ac.jp>; ryoga.suzuki.lab@gmail.com <ryoga.suzuki.lab@gmail.com>; yukimasashibata@cibr.ac.cn <yukimasashibata@cibr.ac.cn>; zhangxiaolin@cibr.ac.cn <zhangxiaolin@cibr.ac.cn>; kokimura@nsc.nagoya-cu.ac.jp <kokimura@nsc.nagoya-cu.ac.jp>; 久保田 幸彦(yukubota) <yukubota@fc.ritsumei.ac.jp>; 伊藤 將弘(maito) <maito@sk.ritsumei.ac.jp>; ikuemori@cibr.ac.cn <ikuemori@cibr.ac.cn>
件名: Re: Confirmation of authorship change for PLOS Genetics manuscript PGENETICS-D-26-00144R1

Dear Dr. Moon Sun Jang,

I confirm that I agree with the addition of Xiaolin Zhang as an author of manuscript PGENETICS-D-26-00144R1.

Best,
Hironori J. Matsuyama

--
Hironori J. Matsuyama, Ph.D.
Assistant Professor
Laboratory of Systems Science,
Department of Data Science,
Faculty of Information Engineering,
Toyama Prefectural University, Imizu, Japan.
Email: matsuyam@pu-toyama.ac.jp
Phone: 0766-56-7500 (ext. 3105)

2026/09/22 9:14、nishiwaki@kwansei.ac.jpのメール:

Dear Dr. Moon Sun Jang,

I confirm that I agree with the addition of Xiaolin Zhang as an author of manuscript PGENETICS-D-26-00144R1.

Best,
Kiyoji Nishiwaki

Get Outlook for Android

From: msjang@nsc.nagoya-cu.ac.jp <msjang@nsc.nagoya-cu.ac.jp>
Sent: Monday, 21 September 2026 21:16:21
To: matsuyam@pu-toyama.ac.jp <matsuyam@pu-toyama.ac.jp>;

To: matsuyam@pu-toyama.ac.jp <matsuyam@pu-toyama.ac.jp>;
ryoga.suzuki.lab@gmail.com <ryoga.suzuki.lab@gmail.com>; ＬＡＷ Ｋｉｎｇ Ｃｈｕｅｎ(gr0609hs) <gr0609hs@ed.ritsumei.ac.jp>;
yukimasashibata@cibr.ac.cn <yukimasashibata@cibr.ac.cn>;zhangxiaolin@cibr.ac.cn <zhangxiaolin@cibr.ac.cn>; kokimura@nsc.nagoya-cu.ac.jp <kokimura@nsc.nagoya-cu.ac.jp>; 久保田 幸彦(yukubota) <yukubota@fc.ritsumei.ac.jp>; 伊藤 將弘(maito) <maito@sk.ritsumei.ac.jp>;
nishiwaki@kwansei.ac.jp<nishiwaki@kwansei.ac.jp>;
ikuemori@cibr.ac.cn <ikuemori@cibr.ac.cn>
Cc: msjang@nsc.nagoya-cu.ac.jp <msjang@nsc.nagoya-cu.ac.jp>
Subject: Confirmation of authorship change for PLOS Genetics manuscript PGENETICS-D-26-00144R1

Dear all,

PLOS Genetics has requested written confirmation from all authors regarding the authorship change in our revised manuscript entitled " Tachykinin-like NLP-58 signaling through TKR-1 modulates thermotaxis behavior in Caenorhabditis elegans" (Manuscript ID: PGENETICS-D-26-00144R1).

Xiaolin Zhang has been added as an author based on her contribution to the aged-worm thermotaxis experiments included in the revised manuscript.

If you agree with this authorship change, could you please reply to this email with the following statement:

"I confirm that I agree with the addition of Xiaolin Zhang as an author of manuscript PGENETICS-D-26-00144R1."

Thank you very much for your cooperation.

==============
共著者の皆様

PLOS Geneticsより、改訂に伴う著者変更について、追加著者を含む全著者から
書面による同意確認を提出するよう連絡がありました。

今回の改訂では、加齢線虫を用いた温度走性実験への貢献に基づき、Xiaolin Zhangさんを著者として追加しています。

この著者追加にご同意いただける場合は、本メールへの返信として以下の英文を
そのままお送りいただけますでしょうか。

"I confirm that I agree with the addition of Xiaolin Zhang as an author of manuscript PGENETICS-D-26-00144R1."

PLOSへの提出資料として使用させていただきます。

差出人: **Masahiro ITO** maito@sk.ritsumei.ac.jp
件名: Re: Confirmation of authorship change for PLOS Genetics manuscript PGENETICS-D-26-00144R1
日付: 2026年9月22日 9:09
宛先: Yukimasa Shibata yukimasashibata@cibr.ac.cn
CC: msjang@nsc.nagoya-cu.ac.jp、 matsuyam@pu-toyama.ac.jp、 ryoga.suzuki.lab@gmail.com、 gr0609hs@ed.ritsumei.ac.jp、 zhangxiaolin@cibr.ac.cn、 kokimura@nsc.nagoya-cu.ac.jp、 yukubota@fc.ritsumei.ac.jp、 nishiwaki@kwansei.ac.jp、 ikuemori@cibr.ac.cn

MI

Dear Dr. Moon Sun Jang,

I confirm that I agree with the addition of Xiaolin Zhang as an author
of manuscript PGENETICS-D-26-00144R1.

Best regards,
Masahiro

2026年9月21日(月) 22:36 Yukimasa Shibata <yukimasashibata@cibr.ac.cn>:

> Dear Dr, Moon Sun Jang
>
> I confirm that I agree with the addition of Xiaolin Zhang as an author
> of manuscript PGENETICS-D-26-00144R1.
>
> Best,
>
> Yukimasa Shibata
>
>> -----Original Messages-----
>> From: msjang@nsc.nagoya-cu.ac.jp
>> Send time:Monday, 21/09/2026 20:16:21
>> To: matsuyam@pu-toyama.ac.jp, ryoga.suzuki.lab@gmail.com, gr0609hs@ed.ritsumei.ac.jp, yukimasashibata@cibr.ac.cn, zhangxiaolin@cibr.ac.cn, kokimura@nsc.nagoya-cu.ac.jp, yukubota@fc.ritsumei.ac.jp, maito@sk.ritsumei.ac.jp, nishiwaki@kwansei.ac.jp, ikuemori@cibr.ac.cn
>> Cc: msjang@nsc.nagoya-cu.ac.jp
>> Subject: Confirmation of authorship change for PLOS Genetics manuscript PGENETICS-D-26-00144R1
>>
>> Dear all,
>>
>> PLOS Genetics has requested written confirmation from all authors regarding the authorship change in our revised manuscript entitled “ Tachykinin-like NLP-58 signaling through TKR-1 modulates thermotaxis behavior in Caenorhabditis elegans” (Manuscript ID: PGENETICS-D-26-00144R1).
>>
>> Xiaolin Zhang has been added as an author based on her contribution to the aged-worm thermotaxis experiments included in the revised manuscript.
>>
>> If you agree with this authorship change, could you please reply to this email with the following statement:
>>
>> “I confirm that I agree with the addition of Xiaolin Zhang as an author of manuscript PGENETICS-D-26-00144R1.”
>>
>> Thank you very much for your cooperation.
>>
>> ==============
>> 共著者の皆様
>>
>> PLOS Geneticsより、改訂に伴う著者変更について、追加著者を含む全著者から書面による同意確認を提出するよう連絡がありました。
>>
>> 今回の改訂では、加齢線虫を用いた温度走性実験への貢献に基づき、Xiaolin Zhangさんを著者として追加しています。
>>
>> この著者追加にご同意いただける場合は、本メールへの返信として以下の英文をそのままお送りいただけますでしょうか。
>>
>> “I confirm that I agree with the addition of Xiaolin Zhang as an author of manuscript PGENETICS-D-26-00144R1.”
>>
>> PLOSへの提出資料として使用させていただきます。
>>
>> お忙しいところ恐れ入りますが、よろしくお願いいたします。
>>
>> Best regards,
>> Moon Sun Jang

--
Masahiro ITO, Ph.D.
Professor
Department of Bioinformatics,
Ritsumeikan University
1-1-1 Nojihigashi, Kusatsu, Shiga 525-8577, Japan

差出人: nishiwaki@kwansei.ac.jp
件名: RE: Confirmation of authorship change for PLOS Genetics manuscript PGENETICS-D-26-00144R1
日付: 2026年9月22日 9:14
宛先: gr0609hs@ed.ritsumei.ac.jp、 msjang@nsc.nagoya-cu.ac.jp、 matsuyam@pu-toyama.ac.jp、 ryoga.suzuki.lab@gmail.com、 yukimasashibata@cibr.ac.cn、 zhangxiaolin@cibr.ac.cn、 kokimura@nsc.nagoya-cu.ac.jp、 yukubota@fc.ritsumei.ac.jp、 ITO MASAHIRO maito@sk.ritsumei.ac.jp、 ikuemori@cibr.ac.cn
CC: msjang@nsc.nagoya-cu.ac.jp

N

Dear Dr. Moon Sun Jang,

I confirm that I agree with the addition of Xiaolin Zhang as an author of manuscript PGENETICS-D-26-00144R1.

Best,
Kiyoji Nishiwaki

Get [Outlook for Android]

From: msjang@nsc.nagoya-cu.ac.jp <msjang@nsc.nagoya-cu.ac.jp>
Sent: Monday, 21 September 2026 21:16:21
To: matsuyam@pu-toyama.ac.jp <matsuyam@pu-toyama.ac.jp>; ryoga.suzuki.lab@gmail.com <ryoga.suzuki.lab@gmail.com>; ＬＡＷ Ｋｉｎｇ Ｃｈｕｅｎ (gr0609hs) <gr0609hs@ed.ritsumei.ac.jp>; yukimasashibata@cibr.ac.cn <yukimasashibata@cibr.ac.cn>; zhangxiaolin@cibr.ac.cn <zhangxiaolin@cibr.ac.cn>; kokimura@nsc.nagoya-cu.ac.jp <kokimura@nsc.nagoya-cu.ac.jp>; 久保田 幸彦(yukubota) <yukubota@fc.ritsumei.ac.jp>; 伊藤 將弘(maito) <maito@sk.ritsumei.ac.jp>; nishiwaki@kwansei.ac.jp <nishiwaki@kwansei.ac.jp>; ikuemori@cibr.ac.cn <ikuemori@cibr.ac.cn>
Cc: msjang@nsc.nagoya-cu.ac.jp <msjang@nsc.nagoya-cu.ac.jp>
Subject: Confirmation of authorship change for PLOS Genetics manuscript PGENETICS-D-26-00144R1

Dear all,

PLOS Genetics has requested written confirmation from all authors regarding the authorship change in our revised manuscript entitled " Tachykinin-like NLP-58 signaling through TKR-1 modulates thermotaxis behavior in Caenorhabditis elegans" (Manuscript ID: PGENETICS-D-26-00144R1).

Xiaolin Zhang has been added as an author based on her contribution to the aged-worm thermotaxis experiments included in the revised manuscript.

If you agree with this authorship change, could you please reply to this email with the following statement:

"I confirm that I agree with the addition of Xiaolin Zhang as an author of manuscript PGENETICS-D-26-00144R1."

Thank you very much for your cooperation.

==============
共著者の皆様

PLOS Geneticsより、改訂に伴う著者変更について、追加著者を含む全著者から

PLOS Geneticsより、改訂に伴う著者変更について、追加著者を含む全著者から
書面による同意確認を提出するよう連絡がありました。

今回の改訂では、加齢線虫を用いた温度走性実験への貢献に基づき、Xiaolin Zhangさんを著者として追加しています。

この著者追加にご同意いただける場合は、本メールへの返信として以下の英文をそのままお送りいただけますでしょうか。

“I confirm that I agree with the addition of Xiaolin Zhang as an author of manuscript PGENETICS-D-26-00144R1.”

PLOSへの提出資料として使用させていただきます。

お忙しいところ恐れ入りますが、よろしくお願いいたします。

Best regards,
Moon Sun Jang

差出人: **Ikue Mori** ikuemori@cibr.ac.cn
件名: Re: Confirmation of authorship change for PLOS Genetics manuscript PGENETICS-D-26-00144R1
日付: 2026年9月22日 11:15
宛先: msjang@nsc.nagoya-cu.ac.jp
CC: matsuyam@pu-toyama.ac.jp、 ryoga.suzuki.lab@gmail.com、 gr0609hs@ed.ritsumei.ac.jp、 yukimasashibata@cibr.ac.cn、 zhangxiaolin@cibr.ac.cn、 kokimura@nsc.nagoya-cu.ac.jp、 yukubota@fc.ritsumei.ac.jp、 maito@sk.ritsumei.ac.jp、 nishiwaki@kwansei.ac.jp

---

Dear Dr. Moon Sun Jang

I confirm that I agree with the addition of Xiaolin Zhang as an author of manuscript PGENETICS-D-26-00144R1.

With bet regards and wishes,
Ikue

> -----Original Messages-----
> From: msjang@nsc.nagoya-cu.ac.jp
> Send time:Monday, 21/09/2026 20:16:21
> To: matsuyam@pu-toyama.ac.jp, ryoga.suzuki.lab@gmail.com, gr0609hs@ed.ritsumei.ac.jp, yukimasashibata@cibr.ac.cn, zhangxiaolin@cibr.ac.cn, kokimura@nsc.nagoya-cu.ac.jp, yukubota@fc.ritsumei.ac.jp, maito@sk.ritsumei.ac.jp, nishiwaki@kwansei.ac.jp, ikuemori@cibr.ac.cn
> Cc: msjang@nsc.nagoya-cu.ac.jp
> Subject: Confirmation of authorship change for PLOS Genetics manuscript PGENETICS-D-26-00144R1
>
> Dear all,
>
> PLOS Genetics has requested written confirmation from all authors regarding the authorship change in our revised manuscript entitled "Tachykinin-like NLP-58 signaling through TKR-1 modulates thermotaxis behavior in Caenorhabditis elegans" (Manuscript ID: PGENETICS-D-26-00144R1).
>
> Xiaolin Zhang has been added as an author based on her contribution to the aged-worm thermotaxis experiments included in the revised manuscript.
>
> If you agree with this authorship change, could you please reply to this email with the following statement:
>
> "I confirm that I agree with the addition of Xiaolin Zhang as an author of manuscript PGENETICS-D-26-00144R1."
>
> Thank you very much for your cooperation.
>
> ==============
> 共著者の皆様
>
> PLOS Geneticsより、改訂に伴う著者変更について、追加著者を含む全著者から書面による同意確認を提出するよう連絡がありました。
>
> 今回の改訂では、加齢線虫を用いた温度走性実験への貢献に基づき、Xiaolin Zhangさんを著者として追加しています。
>
> この著者追加にご同意いただける場合は、本メールへの返信として以下の英文をそのままお送りいただけますでしょうか。
>
> "I confirm that I agree with the addition of Xiaolin Zhang as an author of manuscript PGENETICS-D-26-00144R1."
>
> PLOSへの提出資料として使用させていただきます。
>
> お忙しいところ恐れ入りますが、よろしくお願いいたします。
>
> Best regards,
> Moon Sun Jang

<><><><><>--
Ikue Mori, PhD
Distinguished Investigator
Chinese Institute for Brain Research, Beijing (CIBR)
Beijing, China

Former and Founding Director
Neuroscience Institute, Graduate School of Science
Professor Emeritus of Nagoya University
Nagoya University
Nagoya, Japan
--<><><><><>
